# The deubiquitinase USP7 promotes HNSCC progression via deubiquitinating and stabilizing TAZ

**DOI:** 10.1038/s41419-022-05113-z

**Published:** 2022-08-05

**Authors:** Jin Li, Yibin Dai, Han Ge, Songsong Guo, Wei Zhang, Yanling Wang, Laikui Liu, Jie Cheng, Hongbing Jiang

**Affiliations:** 1 Department of Oral and Maxillofacial Surgery, The Affiliated Stomatological Hospital, Nanjing Medical University, Nanjing, 210029 Jiangsu People’s Republic of China; 2 Jiangsu Key Laboratory of Oral Disease, Nanjing Medical University, Nanjing, 210029 Jiangsu People’s Republic of China; 3 Jiangsu Province Engineering Research Center of Stomatological Translational Medicine, Nanjing Medical University, Nanjing, 210029 Jiangsu People’s Republic of China

**Keywords:** Head and neck cancer, Ubiquitylated proteins

## Abstract

Dysregulated abundance, location and transcriptional output of Hippo signaling effector TAZ have been increasingly linked to human cancers including head neck squamous cell carcinoma (HNSCC). TAZ is subjected to ubiquitination and degradation mediated by E3 ligase β-TRCP. However, the deubiquitinating enzymes and mechanisms responsible for its protein stability remain underexplored. Here, we exploited customized deubiquitinases siRNA and cDNA library screen strategies and identified USP7 as a bona fide TAZ deubiquitinase in HNSCC. USP7 promoted cell proliferation, migration, invasion in vitro and tumor growth by stabilizing TAZ. Mechanistically, USP7 interacted with, deubiquitinated and stabilized TAZ by selectively removing its K48-linked ubiquitination chain independent of canonical Hippo kinase cascade. USP7 potently antagonized β-TRCP-mediated ubiquitin-proteasomal degradation of TAZ and enhanced its nuclear retention and transcriptional output. Importantly, overexpression of USP7 correlated with TAZ upregulation, tumor aggressiveness and unfavorable prognosis in HNSCC patients. Pharmacological inhibition of USP7 significantly suppressed tumor growth in both xenograft and PDX models. Collectively, these findings identify USP7 as an essential regulator of TAZ and define USP7-TAZ signaling axis as a novel biomarker and potential therapeutic target for HNSCC.

## Introduction

Head neck squamous cell carcinoma (HNSCC) is the sixth most common malignancy worldwide, posing great medical and socioeconomic challenges [1]. Conventional therapeutic strategies for this devastating malignancy include ablative surgery, chemotherapy, radiotherapy and EGFR-targeting therapies [2–4]. However, long-term survival in patients with advanced diseases remains unfavorable. Moreover, the paucity of robust diagnostic/prognostic biomarkers and therapeutic targets further complicates the progress of treatment outcomes. These difficulties highlight the urgent need to identify potent translational biomarkers and therapeutic targets to improve clinical management of HNSCC [5].

The evolutionarily conserved Hippo signaling has been increasingly appreciated as key players underlying cell proliferation and growth, apoptosis as well as epithelial-to-mesenchymal transition. When it goes awry, aberrant Hippo signaling usually results in multiple human cancers including HNSCC [6, 7]. Transcriptional coactivator with PDZ-binding motif (TAZ, also known as WWTR1) and its paralog Yes-associated protein (YAP) are two well-established Hippo downstream effectors across a myriad of physiological and pathological settings, which are under tight regulations by the Hippo core kinase cascades such as mammalian Ste20-like (MST) kinases 1/2 and large tumor suppressors (LATS) 1/2. These kinases cooperatively phosphorylate TAZ/YAP, leading to their cytoplasmic retention and subsequent degradation by ubiquitin-proteasome system [8]. In most cancers, unphosphorylated activated TAZ/YAP translocate into nucleus, which in turn binds with multiple transcriptional factors including TEAD1-4 to activate transcription of downstream targets to induce unstrained proliferation, metastasis, chemoresistance and cancer stem cell features, thus making them attractive therapeutic targets [8–10]. Previous studies have established that the abundance, cytoplasmic-nuclear location and biological activities of TAZ/YAP proteins are modulated by multiple post-translational modifications like phosphorylation [11–14], ubiquitination [15–17] or methylation [18] under specific physiopathological contexts. These findings have opened avenues that therapeutically targeting these modifications of TAZ/YAP might be a viable anti-cancer strategy with translational promises in cancers harboring hyperactivated TAZ/YAP.

Ubiquitination and deubiquitination, two antagonistic biological processes executed by E1–E3 ubiquitin ligase and deubiquitinase (DUB), are intricately involved in the stability and hemostasis of most proteins under physiopathological settings [19, 20]. Disrupted balance of these two processes usually results in overexpression of oncogenic proteins or repression of tumor suppressors to facilitate tumorigenesis [21]. For example, ubiquitin-specific protease 7 (USP7, also known as HAUSP) has been reported to stabilize several substrates including p53 [22], MDM2 [22, 23], N-Myc [24], HIF-1α [25] and PHF8 [26] to be critically involved in cancer initiation and progression. Noticeably, several chemical compounds have been identified to selectively target USP7 with remarkable anti-cancer effects in preclinical cancer models [27–30]. Pioneering works have documented that the protein abundance and stability of TAZ are largely dictated by its ubiquitination and degradation by SCF^β-TRCP^ E3 ubiquitin ligase [12, 14]. However, specific DUB candidates for TAZ in HNSCC have been underexplored until now.

Here, we initially exploited the customized DUBs siRNA and cDNA libraries to screen DUB candidates for TAZ and identified USP7 as a potent interacting DUB for TAZ stability and nuclear retention. Furthermore, our results from in vitro cellular assays, preclinical animal models and clinical samples revealed oncogenic USP7-TAZ axis as robust biomarkers and therapeutic targets for HNSCC.

## Materials and methods

### Cell culture, transfection and chemicals

A panel of cell lines Cal27, Fadu and HEK293T obtained from ATCC was authenticated by short tandem repeat (STR) profiling and routinely tested negative for *mycoplasma* contamination at regular intervals. HNSCC cells and HEK293T cells were cultured in DMEM/F12 or DMEM (Thermo, USA) both supplemented with 10% FBS (Thermo). Cells were cultured in a constant temperature and humidity atmosphere (37 °C and 5% CO_2_). Cell transfection was performed by Lipofectamine™ 2000 (Thermo) according to the manufacturer’s protocols. Chemical compounds and recombinant cytokines were purchased commercially and their detailed information was listed in Supplementary Table 1.

### DUBs siRNA, cDNA library and shRNA lentivirus

A customized DUBs siRNA library with two independent siRNAs targeting individual DUB (Supplementary Table 2) was designed, synthesized and obtained from GenePharma (China). To exclude the potential off-target effects, the oligos targeting human USP7 and DCAF12 were designed and utilized in selected experiments. The sequence with the most potency of knockdown against USP7 (siUSP7-3) was subcloned into GV248 lentivirus vector (hU6-MCS-Ubiquitin-EGFP-IRES-puromycin) which was subsequently subjected to routine lentivirus production (named as shUSP7). Cal27 and Fadu cells were infected with shUSP7 lentivirus in the presence of 5 μg/ml Polybrene and were further selected with puromycin (5 μg/ml) for 1 week. The efficiency of siRNA- or shRNA-mediated knockdown was verified by qRT-PCR and immune blot, respectively. Detailed sequences of siRNA/shRNA targeting USP7 were listed in Supplementary Table 3.

DUBs cDNA plasmid library (Supplementary Table 4) and pRK5-HA-Ubiquitin-WT (HA-Ub-WT, #17608), pRK5-HA-Ubiquitin-K48 (HA-Ub-K48only, #17605) and pRK5-HA-Ubiquitin-K63 (HA-Ub-K63only, #17606) were purchased from Addgene (USA). pCAN-Myc-USP7-WT-Neo and pCAN-Myc-USP7-C223S-Neo were the generous gifts from Prof. Lori Frappier. Myc-TAZ, Myc-β-TRCP, Myc-MST2, Myc-USP7 (1-208aa), Myc-USP7 (208-560aa) and Myc-USP7 (560-1102aa) were generated by subcloning human corresponding cDNA into pcDNA3.1-Myc vector via BamHI sites, respectively. V5-LATS2, Flag-TAZ, Flag-TAZ (1-125aa), Flag-TAZ (125-270aa) and Flag-TAZ (270-400aa) cDNA were subcloned into pcDNA3.1+ via BamHI sites. TAZ mutant construct Flag-TAZ^4SA^ (S66A, S89A, S117A and S311A) constructed via Phusion Site-directed Mutagenesis Kit (F541, Thermo). These vectors were verified by direct sequencing before use.

### DUB screen for TAZ stability

For DUB screen for TAZ, individual siRNA in the DUB siRNA library was transiently transfected into Cal27/Fadu cells at a final concentration of 100 nM. Cells were harvested at 72-h time point for western blot assay for TAZ detection. Complementarily, equivalent concentrations (1 μg/ml) of Flag-HA-tagged DUB plasmids in the DUB cDNA library were introduced into HEK293T cells. Forty-eight hours after transfection, cells were lysed and subjected to western blot assay for the measurements of endogenous TAZ protein abundance.

### Subcellular protein fractionation and western blot

Extraction of cytoplasmic and nuclear proteins was performed using Nuclear and Cytoplasmic Extraction Reagents Kit (78833, Thermo) according to the user’s manual. For western blot analysis, cells were lysed in cold lysis buffer supplemented with protease inhibitor cocktail. Protein concentration was determined by Bradford method and equal amounts of proteins were loaded and resolved in 5–10% SDS-PAGE gels. PVDF membranes were blocked with 5% (wt/vol) nonfat dry milk in Tris-buffered saline with Tween-20, incubated with indicated primary antibodies followed by HRP-conjugated secondary antibodies, and finally detected using on SuperSignal West Pico reagents (34577, Thermo). Relevant primary antibodies used were listed in Supplementary Table 5.

### Immunoprecipitation (IP) and deubiquitination assay

Cells were lysed with 1 ml IP lysis buffer (P0013, Beyotime) containing protease inhibitors for 15 min at 4 °C. After the protein concentrations were measured, equal amounts of lysates were used for immunoprecipitation. Immunoprecipitation was performed with antibodies and Pierce™ Protein A/G Plus Agarose (20423, Thermo) overnight at 4 °C. Then, the precipitants were washed four times with IP lysis buffer, and the immune complexes were eluted with sample buffer containing 1% SDS for 5 min at 95 °C. Both immunoprecipitated proteins and total lysates were resolved by SDS-PAGE and immunoblot analyses. Relevant primary antibodies used were listed in Supplementary Table 5.

For in vivo deubiquitination assay, cells were treated with 10 μM MG-132 for 8 h and homogenized with IP lysis buffer (supplemented with 10 μM MG-132). Cell lysates were incubated with indicated antibodies overnight at 4 °C. Pierce™ Protein A/G Plus Agarose were added, and 2 h later the beads were washed five times with IP buffer and then subjected to western blot.

### In vitro binding assay

To generate and purify Flag-TAZ/Myc-USP7-WT recombined proteins from mammal cells, HEK293T cells were transfected with the indicate plasmid and cultured in SFM4Transfx-293 without L-Glutamine culture medium (SH30860.02, Thermo) for another 4 days. Cells were harvested and the recombined proteins were purified by using Pierce™ anti- DYKDDDDK Magnetic Agarose (A36797, Thermo)/ Pierce™ Anti-c-Myc Agarose (20168, Thermo) and then eluted with Pierce™ 3×DYKDDDDK peptides (A36805, Thermo)/Pierce™ c-Myc Peptide (20170, Thermo). The purified proteins were verified by SDS-PAGE.

The purified Flag-TAZ and Myc-USP7-WT were mixed with equimolar, incubated (supplemented with protease inhibitor mixture and Pierce™ anti- DYKDDDDK Magnetic Agarose) at 4 °C overnight, and followed by 5-time IP lysis buffer washing step. The magnetic agarose was resuspended and routinely subjected to SDS-PAGE process.

### CHX chase assays

In total, 1 × 10^5^ cells were seeded into a Φ6cm culture dish and treated with siRNA/plasmid/inhibitor at a certain time point. Four days post seeding, cells were treated with CHX (100 μg/ml) and harvested at the indicated time points for western blot detection.

### Cellular immunofluorescence (IF)

Cells seeded and grown on glass coverslips in a 24-well plate were treated as indicated. Then cells were washed with PBS and fixed with 4% paraformaldehyde in PBS, permeabilized with 0.2% Triton X-100 and blocked with 3% BSA. Cells were further stained with USP7 and TAZ antibodies followed by incubation with fluorescent-dye-conjugated secondary antibodies. Nucleus was counterstained with DAPI (Sigma-Aldrich). Relevant primary antibodies used were listed in Supplementary Table 5.

### RNA-isolation and quantitative real-time PCR (qRT-PCR)

Total RNA was extracted using the MiniBEST Universal RNA Extraction Kit (9767, TaKaRa, Japan) and reversed transcribed into cDNA by PrimeScript™ RT Master Mix Kit (RR036, TaKaRa). Real-time PCR was performed on BI 7900HT Fast Real-Time PCR System (Thermo) with TB Green^®^ Premix Ex Taq™ II (RR820, TaKaRa). 2^−ΔΔCT^ method was used for relative quantification. 18s RNA was used for data normalization. All primer sequences of qRT-PCR were listed in Supplementary Table 6.

### Luciferase reporter assay

The 8×GTIIC-luciferase plasmid was purchased from Addgene (#34615) which contains eight synthetic TEADs binding sites (5′-ACATTCCA-3′). Dual-Luciferase reporter system (A1222, Promega, USA) was used for detecting luciferase reporter activity. After transfection with Myc-USP7-WT/C223S plasmid for 12 h, HEK293T cells were further transfected with the corresponding concentration of Flag-TAZ-WT/4SA or empty control vector, 8×GTIIC-luciferase and pRL-NULL (E227A, Promega) plasmids. Cells were lysed at 36 h and subjected to a plate-reading luminometers for measurement and calculation the Firefly/Renilla ratios.

### CCK-8 and colony formation assays

CCK-8 assay and colony formation assay were performed as we previously reported [31].

### Cell migration and invasion assays

Cell migration and invasion assays were performed as described previously [31]. In brief, cell migration was assessed by wound healing assay, while cell invasion was evaluated by using Costar Transwell™ Permeable Supports 8.0-µm (3422, Corning, USA) with Basement Membrane Extract (BME001, R&D, USA).

### Flow cytometry for apoptosis

Cells were trypsinized with EDTA-free trypsin (15050065, Gbico) and counted. Single-cell suspension with equivalent cell numbers was treated with Annexin V: PE Apoptosis Detection Kit (BD Bioscience, USA) according to the manufacturer’s instructions and analyzed by FACSCanto II (BD Bioscience).

### HNSCC xenograft model and patient-derived xenografts (PDX) model

All animal experiments were complied with animal ethics guidelines and approved by Institutional Animal Care and Use Committee of Nanjing Medical University. In the HNSCC xenograft model, 6-week-old female nu/nu mice would be randomly divided into two groups (at least *n* = 6 per group) and injected with 100 μl cell suspension (2 × 10^6^ Fadu cells with stable infection of shNC/shUSP7) subcutaneously on the both flanks. The longest and shortest diameters of each tumor were measured once every 3 days using vernier calipers. The volume of tumor masses was estimated by the following equation: Volume = (the longest diameter) × (the shortest diameter)^2^/2. Upon the animals being sacrificed, tumor masses were harvested and weighted and then subjected to H&E staining and immunohistochemical (IHC) staining.

In the PDX model, fresh primary HNSCC samples were harvested within 15 min after surgical resection. After careful dissection and removal of necrotic areas, samples were thoroughly rinsed, and trimmed with scissors into 1–2 mm^3^ and then subcutaneously transplanted into the flanks of 5-week female NOD/SCID mice under strict aseptic conditions. The palpable tumor masses appeared at the transplantation sites within 1–3 months and then were transplanted using the same methodology. When the masses volume reached 100 mm^3^, mice were randomly divided into two groups (at least *n* = 6 per group) and labeled. P5091 or vehicle (PBS, control) was administered by intraperitoneal injection (15 mg/kg, tiw.) into tumor-bearing mice. Two weeks later, the tumor masses were harvested for further analyses.

### Clinical specimens and immunohistochemical staining (IHC)

The whole study was approved by Research Ethic Committee of Nanjing Medical University. Written informed consents have been obtained from patients before surgery with regard to clinical samples harvested for experiments. A total number of 99 pathologically diagnosed primary HNSCC samples and 24 paired adjacent non-tumor oral mucosa were obtained between Jan. 2012 to Dec. 2016 at Department of Oral and Maxillofacial Surgery, Affiliated Hospital of Stomatology, Nanjing Medical University.

IHC staining was performed as routine procedures. Briefly, paraformaldehyde-fixed specimens were conventionally dehydrated, paraffin-embedded and partitioned into 4 μm-thick slides. The immunostaining was performed as previously described. The German semi-quantitative 12-point scoring system (percentage score × intensity score) was used to determine the staining results. The detailed information of clinical features and IHC scoring were listed in Table 1. Relevant primary antibodies for IHC were listed in Supplementary Table 5.

**Table 1:** High USP7/TAZ expression and their associations with clinicopathological parameters in 99 patients with HNSCC.

Parameters		USP7		*p* values	TAZ		*p* values
		Low	High		Low	High	
Gender	99	50	49		43	56	
Male	45	23	22	0.9123	20	25	0.9999
Female	54	27	27		23	31	
Age							
<60	44	23	21	0.7530	17	27	0.4206
≥60	55	27	28		26	29	
Smoking							
No	61	31	30	0.9368	26	35	0.8384
Yes	38	19	19		17	21	
Alcohol use							
No	61	30	31	0.7384	25	36	0.5405
Yes	38	20	18		18	20	
Tumor size							
T1–T2	61	40	21	**0.0002**	32	29	**0.0239**
T3–T4	38	10	28		11	27	
Pathological grade							
I	72	33	39	0.1290	29	43	0.3648
II–III	27	17	10		14	13	
Cervical node metastasis							
N(0)	57	38	19	**0.0002**	30	27	**0.0407**
N(+)	42	12	30		13	29	
Clinical stage							
I–II	44	34	10	**<0.0001**	28	16	**0.0005**
III–IV	55	16	39		15	40	

Associations between USP7/TAZ protein expression and clinicopathological parameters in primary HNSCC samples.

Bold values indicates statistically significant at *P* < 0.05.

The Chi-squared test was applied for USP7 and TAZ expression and various clinicopathological parameters. Differences were considered statistically (bold values) significant at *P* < 0.05.

### Bioinformatics analyses

TAZ mRNA expression in TCGA Pan-cancer and positively correlated genes of USP7 and TAZ in HNSCC were downloaded from cBioPortal (https://www.cbioportal.org/). The overlapped genes between USP7 and TAZ correlated genes were ranked according to the relatedness (high to low) and then subjected to Gene Ontology (GO, using ClusterProfiler [32], vison 4.0.5) and Kyoto Encyclopedia of Genes and Genomes (KEGG, using ClusterProfiler). Gene expression data of 499 TCGA-HNSC cancer samples were downloaded from Genomic Data Commons (https://portal.gdc.cancer.gov) and normalized via TPM method. The upper-twentieth (*n* = 100) with the highest levels of USP7 expression and lower-20th (*n* = 100) with the lowest levels of USP7 expression samples were picked up and subjected to differential analyses (limma, version 3.46.0). In total, 9478 differentially expressed genes (DEGs) (|logFC | ≥ 1 and adjust *p* < 0.05) were ranked and analyzed by Gene set enrichment analysis (GSEA) using ClusterProfiler. Custom gene signature scores were calculated via ssGSEA (GSVA, version 1.441).

### Statistical analysis

All quantitative data were presented as mean ± SD which were mostly originated from three independent experiments. The quantitative data were statistically analyzed with Student’s *t* test or ANOVA with post hoc test by GraphPad Prism 8.0 as indicated. The Chi-squared and Fisher exact tests were applied for TAZ/USP7 expression and various clinicopathological parameters. The Kaplan–Meier method and Log-rank test were used for the assessment of patient survival. Differences were supposed with statistical significance at *p* < 0.05 (*) and *p* < 0.01 (**).

## Results

### Identification of USP7 as a deubiquitinase for TAZ

**Fig. 1:**
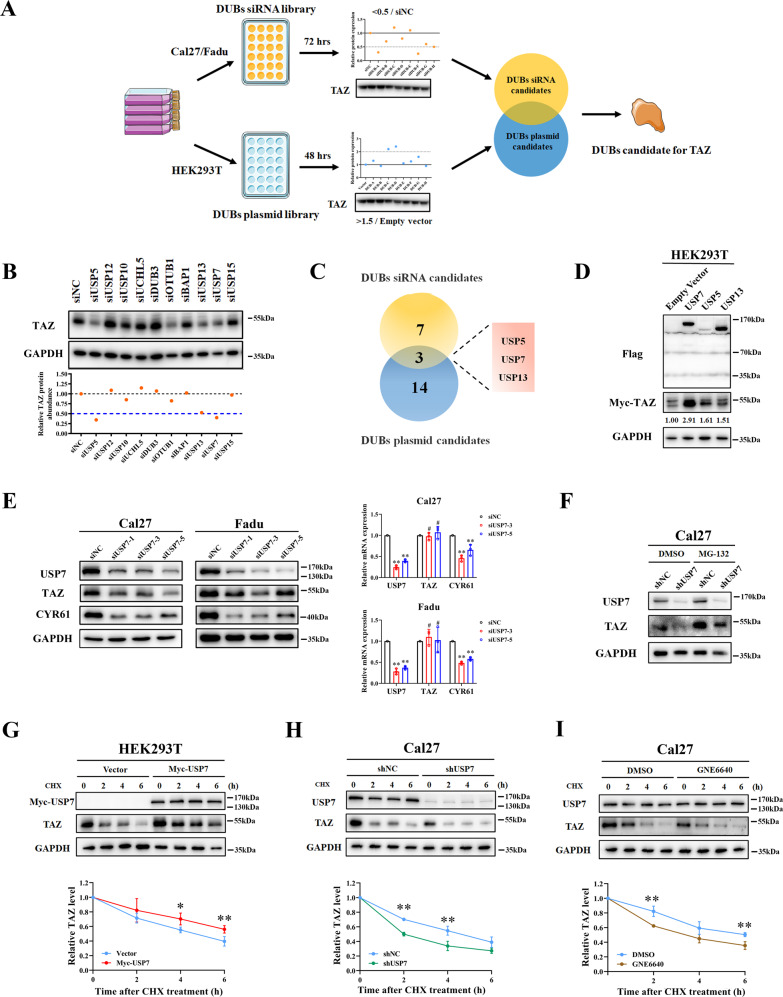
The DUBs screen identifies USP7 as a potent regulator of TAZ stability. **A** Schematic diagram depicting the experimental design for DUBs screens to identify DUB for TAZ. **B** The protein abundance of endogenous TAZ was determined by western blot following transient transfection of siRNAs targeting selected DUBs into Cal27 (72 h). **C** The Venn diagram indicated three overlapped DUB candidates (USP5, USP7 and USP13) identified from both DUBs siRNA and cDNA plasmid screen. **D** Myc-tagged TAZ cDNA plasmid was co-transfected with individual USP5, USP7 and USP13 cDNA plasmids into HEK293T cells. Whole cell lysates were subjected to western blot assay after 48 h transfection. **E** The protein abundance of TAZ and its downstream target CYR61 were significantly reduced upon transfection of three independent siRNAs targeting human USP7 in Cal27 and Fadu cells as measured by western blot (left panel). qRT-PCR data revealed no obvious changes of TAZ mRNA after USP7 knockdown (right panel). **F** TAZ protein abundance was partly rescued after MG-132 (10 μM, 8 h) treatment after USP7 knockdown. **G** The half-life of TAZ protein was markedly elongated following ectopic overexpression of USP7 in HEK293T cells as gauged in the CHX chase assay Cell were treated with cycloheximide (CHX, 100 μg/ml) and harvested for western blot at the indicated time points. **H** The half-life of TAZ protein was significantly shortened after endogenous USP7 knockdown in Cal27 cells as gauged in the CHX chase assay. **I** The half-life of TAZ protein was significantly shortened after treatment of USP7 chemical inhibitor GNE6640 (10 μM) in Cal27 cells as gauged in the CHX chase assay. Data were presented as mean ± SD from three independent experiments. *p < 0.05, **p < 0.01.

Our previous studies and others have established TAZ as a key oncogenic mediator underlying tumorigenesis across multiple cancer contexts including HNSCC [10, 31, 33]. However, pan-cancer examination of genetic alternations and mRNA expression of TAZ from The Cancer Genome Atlas (TCGA) database revealed rare frequencies of gains, loss and mutation as well as considerable variations in transcriptional abundance across most cancers (Supplementary Fig. 1). Moreover, delicate turnover of TAZ/YAP is intricately subjected to various post-translational modifications including phosphorylation and ubiquitination [11, 14]. Therefore, we reasoned that aberrant overexpression of TAZ protein in HNSCC might be largely due to disturbed balance between its production and degradation. To address this, we initially validated that the TAZ protein was unstable in HNSCC cells and its stability was regulated by proteasome-mediated degradation as evidenced that TAZ was significantly stabilized upon proteasome inhibitors treatments (Supplementary Fig. 2). These results encouraged us to explore the DUB candidates responsible for TAZ stability in HNSCC. We exploited two independent, unbiased in vitro screen approaches including a customized siRNA library consisting of two non-overlapping siRNA targeting 56 known DUBs and a small selected DUB cDNA overexpressing library (Fig. 1A). Following DUB siRNA or cDNA plasmids transfection, the abundance of endogenous TAZ protein was examined by western blot. Our complementary approaches have identified USP5, USP7 and USP13 as top DUB candidates for TAZ protein stability in HNSCC (Fig. 1B, C and Supplementary Figs. 3 and 4). We transfected Flag-tagged USP5, USP7 and USP13 cDNA plasmids into HEK293T cells and validated that they enhanced expression of endogenous TAZ (Fig. 1D). We focused on USP7 as a putative DUB for TAZ in the following experiments as it stood out as the most potent candidate affecting TAZ protein. Next, we performed independent experiments using another three siRNAs targeting USP7 and found that TAZ and its well-established downstream target CYR61 were both significantly reduced upon siUSP7 treatments (Fig. 1E, left panel). However, the mRNA levels of TAZ remained largely unaltered after USP7 knockdown, consistent with the notion that USP7 might promote TAZ at the post-translational level (Fig. 1E, right panel). Noticeably, TAZ reduction induced by USP7 knockdown was profoundly attenuated by MG-132 treatment (Fig. 1F). The steady-state abundance of TAZ protein was markedly increased upon enforced USP7 overexpression in a dose-dependent manner (Supplementary Fig. 5A). Transcriptional activities of TAZ-TEAD were impaired following USP7 knockdown as gauged by 8×GTIIC-luciferase reporter assays (Supplementary Fig. 5B). Moreover, small-molecule inhibitor of USP7 P5091 [27] and GNE6640 [29] significantly reduced TAZ protein but not its mRNA expression in vitro (Supplementary Fig. 5C, D). TAZ stability was potently enhanced upon enforced USP7 overexpression in vitro (Fig. 1G and Supplementary Fig. 5E). In contrast, TAZ half-life was markedly decreased when endogenous USP7 was knocked down or its activities were selectively inhibited by P5091 or GNE6640 (Fig. 1H, I and Supplementary Fig. 5F, G). Taken together, our DUB screens identified USP7 as a putative DUB candidate to stabilize TAZ protein via attenuating its proteasomal degradation in HNSCC.

### USP7 promotes HNSCC progression via TAZ

**Fig. 2:**
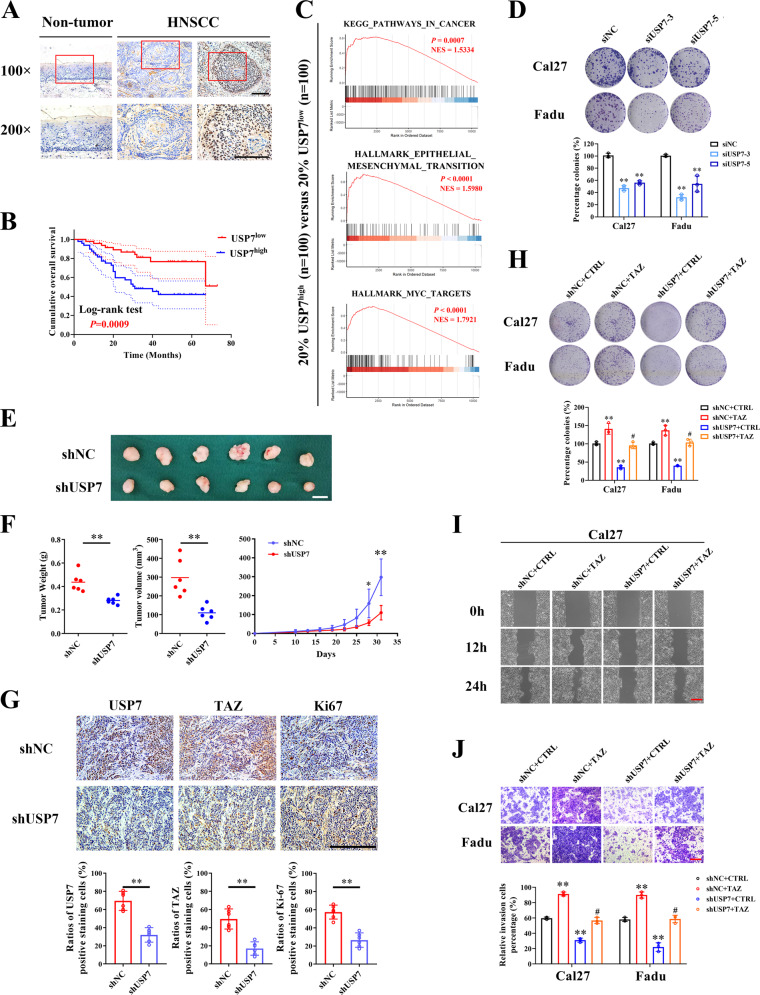
USP7 promotes HNSCC progression by TAZ. **A** Representative images of USP7 expression in human normal oral mucosa and primary HNSCC specimens via immunohistochemical staining. Scale bar: 100 μm. **B** Patients with HNSCC were stratified with USP7 IHC score (low or high). Kaplan–Meier plots revealed that patients with USP7 high-staining had much lower survival rates as compared to those with low USP7 staining (Log-rank test). **C** Gene set enrichment analysis (GSEA) from patients in TCGA-HNSC with higher expression of USP7 (*n* = 100) compared to those with lower expression of USP7 (*n* = 100) suggested that USP7 associated multiple oncogenic properties. **D** The potentials of colony formation were significantly impaired in USP7 knockdown cells. Quantifications were shown in the low panel. **E**, **F** Fadu cells infected with shUSP7 or shNC (non-targeting control) lentivirus were inoculated in both flanks of immunodeficient nude mice (*n* = 6 animals per group). Tumor masses were monitored in regular intervals and finally harvested after animals sacrifice at day 32 (**E**). USP7 knockdown impaired tumor growth in vivo as measured by tumor volume and weights (**F**). Scale bar: 1 cm. **G** Representative IHC staining of USP7, TAZ and Ki-67 in the same region of samples from xenograft tumors (upper panel) and their quantifications data were shown (lower panel). Cell viability and proliferation (**H**), migration (**I**) and invasion (**J**) abilities were significantly reduced following USP7 knockdown but restored by TAZ overexpression as gauged by colony formation assay, wound healing and transwell assays, respectively. *p < 0.05, **p < 0.01.

Although the pro-tumorigenic roles of USP7 have been reported among multiple human cancers, the expression of USP7 and its biological roles in HSNCC remained underexplored. Firstly, we characterized the USP7 expression profile in HNSCC via analyzing the publicly available datasets. USP7 mRNA was aberrantly upregulated in HNSCC as compared to its non-tumor counterparts in two independent cohorts (TCGA-HNSC OSCC samples subset, named TCGA-OSCC and GSE25093, Supplementary Fig. 6A). Subsequently, we performed IHC staining of USP7 in 99 primary HNSCC samples to detect its abundance and subcellular location. As shown in Fig. 2A, positive nuclear staining of USP7 was detected in HNSCC samples while much less positive staining was observed in non-tumor oral mucosa. Quantifications of IHC data revealed that USP7 overexpression is significantly associated with tumor size (*p* = 0.0002), cervical node metastasis (*p* = 0.0002) and clinical stage (*p* < 0.0001) (median values as cut-off, Table 1).

Noticeably, survival analyses further revealed positive associations between USP7 upregulation and unfavorable prognosis in our cohort (*p* = 0.0009, Fig. 2B) and TCGA-HNSC (*p* = 0.0493, Supplementary Fig. 6B). Data from multivariate Cox regression analyses identified USP7 expression as an independent unfavorable prognostic factor after adjusting for multiple clinicopathological parameters (Table 2). Additionally, we classified TCGA-HNSC samples based on USP7 mRNA abundance, derived samples with top and bottom 20% (designated as USP7^high^, USP7^low^) and extracted 9478 DEGs between these two subgroups. GSEA of these DEGs revealed that HNSCC samples with USP7 overexpression harbored genes significantly enriched with cancer-related gene signatures such as Myc-targets, EMT (Fig. 2C) and two previously reported Hippo-YAP/TAZ cancer signatures (Supplementary Fig. 6C) [9, 34].

**Table 2:** Univariate and multivariate Cox regression analyses of USP7 IHC score in this research cohort.

Variables	Univariate analyses		Multivariate analyses	
	HR [95% CI]	*p*	HR [95% CI]	*p*
Combined cohort				
Age (≥60, <60)	1.156 (0.724-1.845)	0.544		
Gender (male, female)	0.788 (0.493-1.26)	0.32		
Smoking history category (≥3, <3)	1.018 (0.634-1.637)	0.94		
Alcohol use (Yes, No)	0.996 (0.615-1.612)	0.987		
Tumor size (T3–T4, T1–T2)	1.843 (1.156-2.94)	**0.010**	0.918 (0.508–1.659)	0.778
Pathological grade (III–IV, I–II)	0.819 (0.2-3.349)	0.781		
Cervical node metastasis (N+, N0)	1.987 (1.235-3.197)	0.005	1.328 (0.765–2.307)	0.313
Clinical stage (III–IV, I–II)	2.499 (1.43-4.366)	**<0.001**	1.749 (0.801–3.82)	0.161
USP7 IHC score (High, Low)	2.361 (1.407-3.963)	**0.001**	1.786 (1.002–3.184)	**0.049**

Univariate and multivariate survival analyses for patients with primary HNSCC.

*HR* hazard ratio, *CI* confidence interval.

Bold values indicates statistically significant at *P* < 0.05.

Univariate and multivariate Cox regression analyses were used for calculating the USP7 IHC score in this research cohort. Differences were considered statistically (bold values) significant at *P* < 0.05.

To further delineate its oncogenic roles, we utilized a siRNA-based loss-of-function approach in vitro. Our results indicated that USP7 knockdown significantly impaired cell proliferation, migration and invasion (Fig. 2D and Supplementary Fig. 7). To substantiate the pro-tumorigenic roles of USP7, we further exploited an HNSCC xenograft model and found that USP7 knockdown reduced tumor growth in vivo, accompanied by diminished TAZ and Ki-67 expression in samples (Fig. 2E–G).

To determine whether TAZ functioned as a key downstream effector of USP7 in HNSCC, we next performed rescue experiments. As shown in Fig. 2H–J and Supplementary Fig. 8A, B, ectopic TAZ overexpression largely abrogated the effects induced by USP7 knockdown including impaired cellular proliferation, migration and invasion. Complementarily, the anti-proliferative effects of GNE6640 were markedly compromised in TAZ knockdown cells (Supplementary Fig. 8C). Moreover, our results from TAZ knockdown revealed negligible effects on USP7 mRNA and protein levels in Cal27 and Fadu cells (data not shown), which suggests that TAZ served as a downstream effector of USP7. In aggregate, our results indicated that UPS7 functioned as a key putative oncogene facilitated HNSCC initiation and progression probably by TAZ and USP7-TAZ axis held potentials as prognostic biomarkers.

### USP7 interacts with and deubiquitinates TAZ

**Fig. 3:**
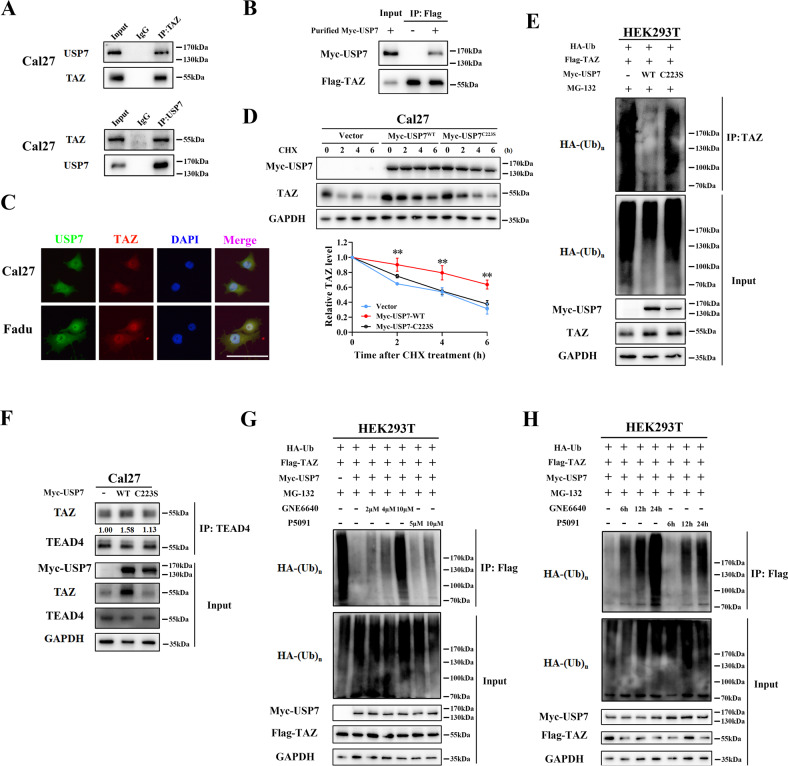
USP7 is a bona fide deubiquitinase for TAZ. **A** USP7 protein associated with TAZ protein. Cal27 cell lysates were subjected to immunoprecipitation with anti-TAZ (upper panel) or anti-USP7 (lower panel) antibody followed by immunoblot with anti-TAZ/anti-USP7 antibody. **B** Purified Flag-TAZ and Myc-USP7 proteins were mixed with equimolar and then subjected to IP assay by anti-Flag magnetic agarose. The binding of Myc-USP7 and Flag-TAZ in vitro were detected by western blot. **C** Cellular immunofluorescence assay shown USP7 (FITC, green) predominantly co-localized with TAZ (Cy3, red) in nucleus in Cal27 and Fadu cells. Scale bar: 50 μm. **D** Myc-tagged USP7 wide-type (USP7^WT^) and its mutant (USP7^C223S^) plasmids were transfected into HEK293T cells which were then subjected to CHX chase assay at the indicated time points. **E** HEK293T cells were co-transfected with HA-Ub with/without Myc-USP7 ^WT^ or Myc-USP7^C223S^ and treated with MG-132 for 8 h before collection. Exogenous TAZ ubiquitination was determined by immunoprecipitation by anti-TAZ antibody and immunoblotted with anti-HA antibody. **F** Myc-tagged USP7 wide-type (USP7^WT^) and its mutant (USP7^C223S^) plasmids were transfected into Cal27 cells for 48 h. The whole cell lysates were subjected to immunoprecipitation by anti-TEAD4 antibody and immunoblotted with anti-TAZ antibody. **G** TAZ ubiquitination was determined in HEK293T cells transfected with indicated plasmids. HEK293T cells were co-transfected with Flag-TAZ, HA-Ub, Myc-USP7. Cells were treated with indicated dosage of GNE6640 (2, 4, 10 μM) or P5091 (5, 10 μM) for 24 h. Then, cell lysates were subjected to immunoprecipitation by anti-Flag antibody and immunoblotted with anti-HA antibody. **H** HEK293T cells were co-transfected with Flag-TAZ, HA-Ub, Myc-USP7. Cells were treated with GNE6640 (10 μM) or P5091 (10 μM) and harvested at indicated time points (6, 12, 24 h). TAZ ubiquitination was detected by IP and western blot, respectively. Data were presented with representative images from three independent experiments. *p < 0.05, **p < 0.01.

Next, we examined whether USP7 could interact with and deubiquitinate TAZ. Reciprocal IP assays detected interactions between endogenous TAZ and USP7 in Cal27 cells (Fig. 3A). Purified Flag-TAZ and Myc-USP7 proteins were pooled in vitro and then immunoprecipitated with anti-Flag antibody. Results suggest the direct interaction between USP7 and TAZ (Fig. 3B). In line with this, the immunofluorescence staining assay showed that USP7 and TAZ were mainly co-localized in nucleus, consistent with previous reports regarding the preferential nuclear location for USP7 and activated TAZ [35, 36] (Fig. 3C).

To determine whether USP7 promoted TAZ stability via deubiquitination, we performed in vivo deubiquitination assay by co-expressing Flag-TAZ, Myc-USP7 and HA-ubiquitin in HEK293T cells. A catalytically inactive USP7 mutant (the 223rd amino acid mutated from cysteine to serine) was generated and transfected into Cal27 cells. As expected, Myc-USP7^C223S^ failed to stabilize TAZ protein as evidenced by minimal changes of endogenous TAZ protein half-life (Fig. 3D) and unaffected mRNA abundance (Supplementary Fig. 9). In Fig. 3E, Myc-USP7^WT^ largely attenuated the TAZ ubiquitination induced exogenous ubiquitin (lane 2) but Myc-USP7^C223S^ did not (lane 3), which implies that the DUB activity of USP7 was required for TAZ stabilization. Complementarily, USP7 inhibitors P5091 and GEN6640 strongly blunted USP7 deubiquitinating activity on TAZ with in both dose-dependent and time-dependent manners (Fig. 3G, H). Moreover, TAZ stabilization by USP7 resulted in increased binding between TAZ and TEAD4, which in turn presumably to execute their functional outputs (Fig. 3F and Supplementary Fig. 5B).

**Fig. 4:**
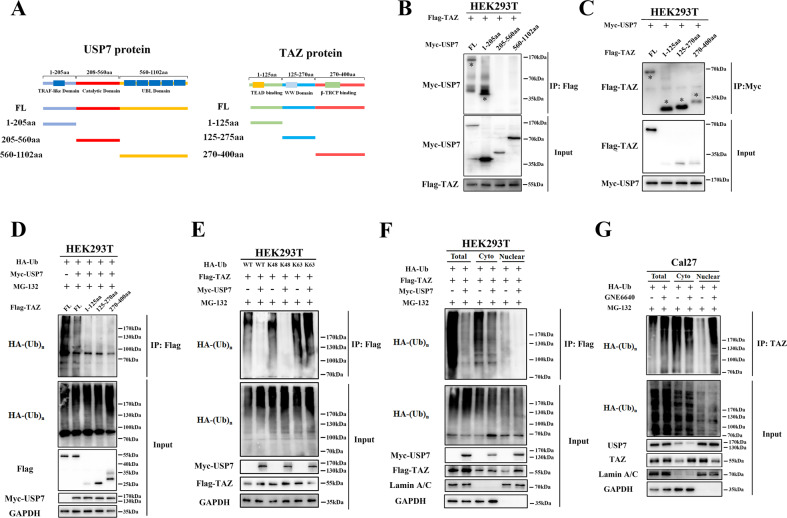
USP7 interacts with TAZ and removes its K48-linked ubiquitin chains. **A** Schematic diagram depicting the USP7 and TAZ protein structure of and their truncation constructs used. **B** Myc-tagged USP7 (full-length or short truncations) and Flag-TAZ plasmids were co-expressed in HEK293T cells. Then cell lysates were subjected to immunoprecipitation by anti-Flag antibody and immunoblotted with anti-Myc antibody. **C** Flag-tagged TAZ (full-length or short truncations) and Myc-USP7 plasmids were co-expressed in HEK293T cells. Then cell lysates were subjected to immunoprecipitation by anti-Myc antibody and immunoblotted with anti-Flag antibody. **D** The ubiquitination of Flag-TAZ or its short truncations was determined in HEK293T cells transfected with indicated plasmids. **E** The ubiquitination linkage of TAZ was determined in HEK293T cells transfected with indicated Flag-TAZ, Myc-USP7, and HA-Ub WT, K48-only, K63-only plasmids. **F** Flag-TAZ, HA-Ub with or without Myc-USP7 plasmids were co-transfected into HEK293 cells. After 48 h, the whole lysates and subcellular fractions (cytoplasmic and nuclear) were subjected to immunoprecipitation by anti-Flag antibody and immunoblotted with anti-HA antibody. **G** Following treatment with MG-132 (10 μM, 8 h) and/or GNE6640 (10 μM, 12 h) in Cal27 cells, the whole lysates and subcellular fractions (cytoplasmic and nuclear) were subjected to immunoprecipitation by anti-TAZ antibody and immunoblotted with anti-HA antibody. Data were presented with representative images from three independent experiments.

As illustrated in Fig. 4A, multiple domains in USP7 and TAZ proteins executed their biological functions by interacting with other partners. To map the regions responsible for USP7 and TAZ interaction, we generated several functional truncations of USP7 (1-205aa, 205-560aa and 560-1102aa) and TAZ (1-125aa, 125-270aa, 270-400aa), respectively. As shown in Fig. 4B, our immunoprecipitation assay revealed that TAZ associated with N-terminal USP7 truncation (1-208aa), but not the other two regions. USP7 associated with full-length of TAZ protein as well as its three truncated mutants (Fig. 4C). To further validate whether USP7 could deubiquitinate these TAZ truncated mutants, we co-expressed Myc-USP7 and Flag-tagged TAZ mutants in HEK293T cells and found that USP7 markedly diminished ubiquitination of these TAZ mutants (Fig. 4D).

Previous reports have delineated that USP7 preferred to cleave ubiquitin linkage at K48 and K63 sites in multiple substrates [29, 37]. To identify the linkage specificity of USP7 in TAZ deubiquitination, we performed the deubiquitination assays by co-expressing HA-Ub-wild type, HA-Ub-K48only or HA-Ub-K63only mutant plasmids with Flag-TAZ and Myc-USP7 in HEK293T cells. As shown in Fig. 4E, USP7 predominantly preferred to cleave K48 polyubiquitin chain on TAZ protein and had minimal effects on K63-linked polyubiquitin chains.

Subsequently, we determined the precise site (cytoplasm or nucleus) whereby USP7 deubiquitinated TAZ. As shown Fig. 4F, USP7 and TAZ overexpressing cDNA plasmids were co-introduced into HEK293T cells and nuclear or cytoplasmic parts from total cellular lysates were fractioned. Ubiquitination accumulation of nuclear TAZ was potently reversed by USP7 overexpression (lane 6), whereas ubiquitination of cytoplasmic TAZ was moderately affected by USP7 (lane 4). In line with this, GNE6640 profoundly impaired USP7-mediated deubiquitination of TAZ in nucleus (Fig. 4G, lane 6). Collectively, our results provided evidence that USP7 was a bona fide TAZ DUB by interaction and ubiquitin cleavage, ultimately promoting TAZ stability and overexpression in HNSCC.

### USP7 and β-TRCP cooperatively maintain TAZ protein abundance in HNSCC

**Fig. 5:**
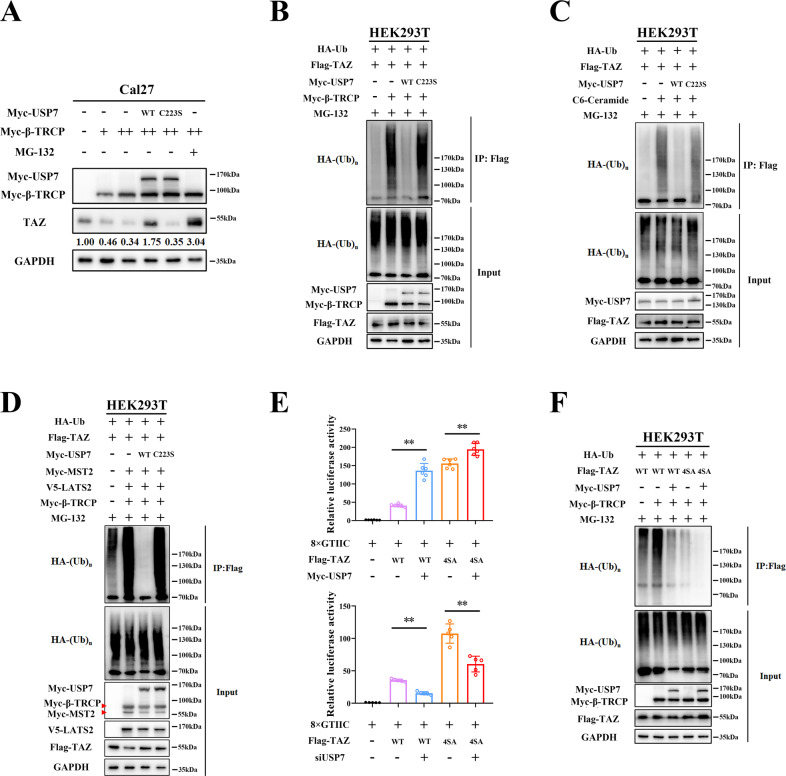
USP7 and β-TRCP cooperatively maintain TAZ overexpression in HNSCC. **A** Endogenous TAZ protein abundance was measured in Cal27 cells transfected with Myc-USP7 (WT/C223S), dosage of β-TRCP (0, 0.5, 1 μg) or treated with MG-132 (10 μM, 8 h). **B** The ubiquitination of TAZ was determined in HEK293T cells transfected with indicated plasmids. HEK293T cells were co-transfected with HA-Ub, Flag-TAZ, Myc-USP7 (WT/C223S) and/or Myc-β-TRCP plasmids. Then cell lysates were subjected to immunoprecipitation by anti-Flag antibody and immunoblotted with anti-HA antibody. **C** The ubiquitination of TAZ was determined in HEK293T cells transfected with indicated plasmids and chemicals. HEK293T cells were co-transfected with HA-Ub, Flag-TAZ, Myc-USP7 (WT/C223S) with/without C6-Ceramide (25 μM, 6 h) treatment. Then cell lysates were subjected to immunoprecipitation by anti-Flag antibody and immunoblotted with anti-HA antibody. **D** The ubiquitination of TAZ was determined in HEK293T cells transfected with indicated plasmids. HEK293T cells were co-transfected with Flag-TAZ, HA-Ub, Myc-MST2, V5-LATS2 and Myc-β-TRCP. Then cell lysates were subjected to immunoprecipitation by anti-Flag antibody and immunoblotted with anti-HA antibody. **E** Luciferase activities on TEAD-binding site (8×GTIIC-luciferase reporter plasmid) were determined in HEK293T cells transfected with Flag-TAZ (WT/4SA), Myc-USP7 plasmids (upper panel) or siUSP7 (lower panel). **F** The ubiquitination of TAZ was determined in HEK293T cells transfected with indicated plasmids. HEK293T cells were co-transfected with Flag-tagged TAZ wide-type, nuclear-localized mutant TAZ^4SA^, Myc-USP7 and Myc-β-TRCP. Cells were further treated with MG-132 (10 μM, 8 h) before collection. Then cell lysates were subjected to immunoprecipitation by anti-Flag antibody and immunoblotted with anti-HA antibody. Data were presented with representative images from three independent experiments. *p < 0.05, **p < 0.01.

It has been well-established that protein ubiquitination and deubiquitination is a tightly regulated balance [21, 38]. Upon these processes were disturbed, various diseases may arise such as cancer. The E3 ligase β-TRCP has been identified to be responsible for TAZ ubiquitination and degradation [14]. We hypothesized that USP7 might cooperate with β-TRCP to maintain TAZ overexpression in HNSCC. As expected, enforced β-TRCP overexpression induced TAZ protein reduction and this effect was largely abrogated by ectopic overexpression of USP7 or MG-132 (Fig. 5A, lane 4, 6). However, USP7^C223S^ mutant failed to reverse this effect induced by β-TRCP (Fig. 5A, lane 5). In parallel, results from ubiquitination assay indicated that ubiquitin aggregation on TAZ was profoundly induced by β-TRCP or reduced by USP7 (Fig. 5B, lane 2, 3). Co-expressed USP7^WT^ but not USP7^C223S^ mutant could potently reverse the β-TRCP-mediated TAZ ubiquitination (Fig. 5B, lane 3, 4). In addition, we utilized C6-ceramide, the chemical compound developed to enhance β-TRCP-mediated YAP/TAZ proteasomal degradation [39] (Supplementary Fig. 10A), to further substantiate the antagonistic actions between β-TRCP and USP7. As shown in Fig. 5C, USP7^WT^ potently abrogated the C6-ceramide-induced TAZ ubiquitination. Previous reports have established that TAZ/YAP were translocated from nucleus to cytoplasmic when cells were grown in high density with Hippo activation [40, 41]. Notably, as shown in Supplementary Fig. 10B, TAZ ubiquitination was significantly increased in high-confluent Cal27 cells compared to that in low-confluent cells (lane 1 versus lane 3). Moreover, USP7 showed higher deubiquitinating activities on TAZ protein in low-density cells (lane 2 versus lane 4).

Given the fact that TAZ ubiquitination and degradation were intricately affected by Hippo core kinases, we next asked whether the interaction between USP7 and TAZ was affected by Hippo signaling. As shown in Supplementary Fig. 10C, D, siRNA-mediated USP7 knockdown had minimal effects on LATS1/2, MST1/2 and YAP/Phospho-YAP (Ser127), thus excluding the possibility that USP7 modulated TAZ modification and abundance via canonical Hippo pathway. Noticeably, as shown in Fig. 5D, activation of Hippo signaling promoted β-TRCP-mediated TAZ ubiquitination whereas exogenous USP7^WT^ but not its mutant potently reversed this effect, thus suggesting that USP7 antagonized β-TRCP in regulation of TAZ ubiquitination. Quite recently, Jin Jiang et al. identified nuclear E3 ubiquitin ligase complex CRL4^DCAF12^ responsible for TAZ/YAP ubiquitination and degradation in addition to β-TRCP [42]. However, our results from siRNA-mediated DCAF12 knockdown failed to support this notion as reflected by minimal changes of TAZ protein (Supplementary Fig. 11).

Consistently, co-expressed USP7 and TAZ constructs robustly induced TEAD-mediated transcriptional activation, whereas USP7 silencing weakened the luciferase activation by TAZ^4SA^ (Fig. 5E). Moreover, we co-expressed USP7, TAZ^WT^ and TAZ^4SA^ mutant into HEK293T cells in the presence of Myc-β-TRCP and found potent deubiquitination of both TAZ^WT^ and TAZ^4SA^ mediated by USP7 (Fig. 5F). Altogether, these findings indicated that TAZ deubiquitination by USP7 was largely independent of canonical Hippo cascade and USP7 cooperates with β-TRCP to maintain protein abundance of TAZ in HNSCC.

### USP7 reduces TAZ nuclear export in HNSCC cells

**Fig. 6:**
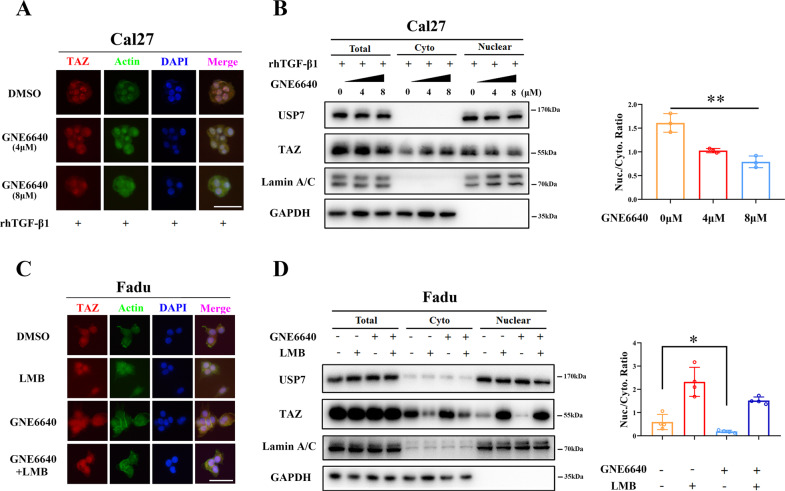
USP7 facilitates TAZ nuclear retention. **A** Pharmacological inhibition of USP7 by GNE6640 significantly reduced nuclear accumulation in an rhTGF-β1-induced TAZ nuclear relocation cell model. Cal27 cells were pretreated rhTGF-β1 (10 ng/ml, 6 h) and then exposed to indicated dose of GNE6640 (0, 4, 8 μM) for another 6 h. TAZ subcellular location (Cy3, red) were determined by immunofluorescence. Nucleus was counterstained with DAPI (blue), and cytoskeleton was visualized with Actin Tracker (FITC, green). Scale bar: 50 μm. **B** The protein abundance of TAZ in cytoplasmic and nuclear fractions was measured by western blot in Cal27 cells treated with rhTGF-β1 (10 ng/ml, 6 h) and GNE6640 (0, 4, 8 μM) for another 6 h. Lamin A/C and GAPDH were utilized as nuclear or cytoplasmic control, respectively. Quantification of results was shown in the right panel. **C** TAZ nuclear export induced GNE6640 (8 μM, 6 h) in Cal27 cells was reversed by Leptomycin B (LMB, 20 ng/ml, 6 h) as gauged by TAZ immunofluorescence. Nucleus was counterstained with DAPI (blue), and cytoskeleton was visualized with Actin Tracker (FITC, green). Scale bar: 50 μm. **D** The protein abundance of TAZ in cytoplasmic and nuclear fractions was measured by western blot in Cal27 cells treated with and GNE6640 (8 μM) and/or LMB (20 ng/ml). Quantification of results was shown in the right panel. Data were presented with representative images from three independent experiments. Student’s *t* test or ANOVA test. *p < 0.05, **p < 0.01.

Accumulating evidence has indicated that nuclear-cytoplasmic shuttle of TAZ intricately links to its activities and biological functions [33, 36]. Reduced nuclear TAZ after pharmacological inhibition of USP7 by GNE6640 (Fig. 4G) prompted us to ask whether USP7 affected TAZ cytoplasmic-nuclear shuttle. Due to the lack of TAZ vectors with preferential nuclear translocation, we utilized the rhTGF-β1-induced TAZ nuclear accumulation cellular model [43]. Initially, the effects of rhTGF-β1 on endogenous TAZ ubiquitination were measured in vitro. As shown in Supplementary Fig. 12A, rhTGF-β1 treatments significantly reduced TAZ ubiquitination and increased its total protein abundance in Cal27 cells. Cal27 cells pretreated with rhTGF-β1 (10 ng/ml, 6 h) followed by GNE6640 for another 6 h resulted in TAZ nuclear export in Cal27 cells (Fig. 6A, B). Moreover, we exploited another cellular model in which Leptomycin B (LMB) blocked TAZ nuclear export [44]. As visualized in Fig. 6C, D, TAZ nuclear export facilitated by GNE6640 was mostly abrogated by LMB in Fadu cells. Notably, LMB exposure moderately reduced TAZ ubiquitination, which was in line with its role as an inhibitor of protein nuclear export (Supplementary Fig. 12B). Taken together, our results suggest that USP7 reduced TAZ nuclear export in HNSCC cells.

### Clinical and translational significance of USP7-TAZ axis in HNSCC

**Fig. 7:**
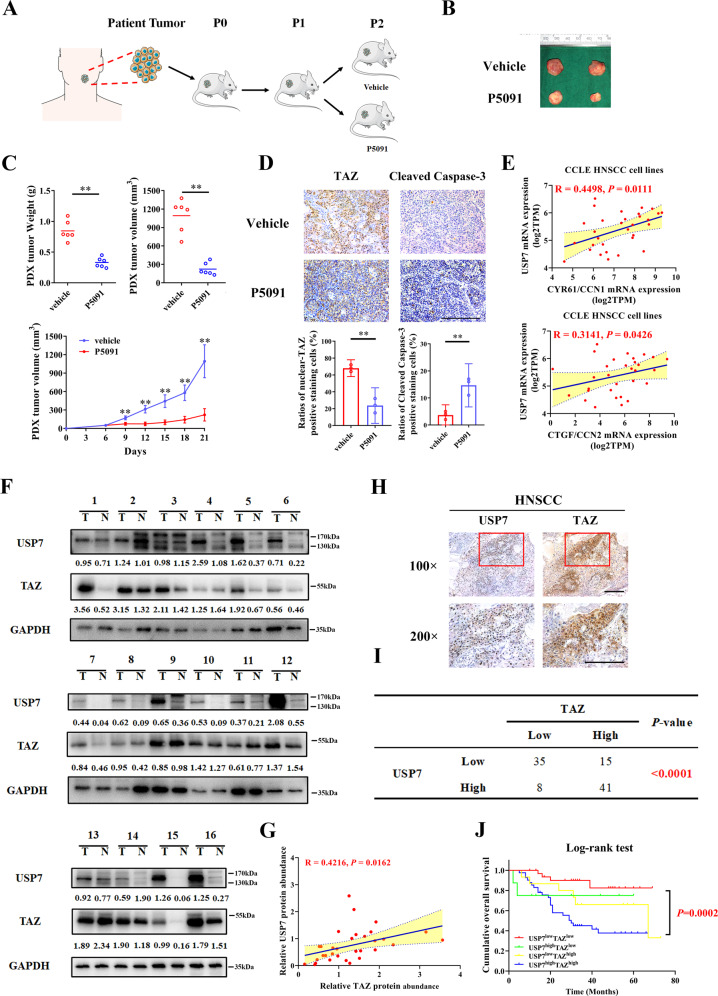
Clinical significance and therapeutic potentials of USP7-TAZ axis in HNSCC. **A** Schematic description of experimental procedures for HNSCC PDX model. Mice bearing human tumor masses with volume over 100 mm^3^ were randomly divided into two groups (*n* = 6 animals per group). Animals were received P5091 (15 mg/kg) or vehicle by peritoneal injection three times per week for 2 consecutive weeks. **B**, **C** Tumor masses in the PDX model were carefully monitored at regular intervals. At Day 21 after initial injection, the masses were harvested for further analyses upon animal sacrifice. Final volume and weight of samples were recorded. **D** Representative IHC staining of TAZ and Cleaved Casepase-3 in PDX samples treated with P5091 or vehicle and their quantification data were shown. Scale bar: 100 μm. Student’s *t* test. **E** The correlation between USP7 and TAZ downstream targets (CYR61/CCN1 and CTGF/CCN2) was assessed. Pearson’s correlation. **F**, **G** The protein expression of USP7 and TAZ in 16 primary HNSCC samples and paired non-cancerous mucosa was detected by western blot (**F**). The correlation between USP7 and TAZ was estimated by Pearson’s correlation (**G**). **H** Representative IHC staining of TAZ and USP7 in primary HNSCC samples. Scale bar: 100 μm. **I** Association between TAZ and USP7 IHC staining in 99 primary HNSCC samples was estimated via Chi-squared test. **J** Patients with concomitant USP7 and TAZ overexpression had the worst survival rates as compared to other patient subgroups as estimated by Kaplan–Meier method and compared with Log-rank test. *p < 0.05, **p < 0.01.

To substantiate the translational potentials of USP7-TAZ axis as novel therapeutic targets, we exploited a HNSCC PDX and P5091 which had been verified as a potent inhibitor of USP7 in preclinical cancer models (Fig. 7A). As shown in Fig. 7B–D, P5091 administration by intraperitoneal injection (15 mg/kg, tiw.) robustly inhibited tumor growth in animals. Noticeably, this therapeutic regime was well-tolerated in animals as evidenced by no obvious loss of body weight and histological changes of a vital organ (data not shown). Additionally, IHC results indicated significantly reduced nuclear staining of TAZ and more cleaved Caspase-3^+^ cancerous cells.

To further delineate the USP7-TAZ axis in clinical samples, we collected fresh HNSCC samples and determined USP7 and TAZ protein abundance by western blot. As shown in Fig. 7F, G, both USP7 and TAZ proteins were significantly upregulated in HNSCC as compared to their non-tumor counterparts. A significant correlation was observed between USP7 and TAZ among these samples. Consistently, we also performed IHC and found that USP7 expression significantly associated with TAZ abundance (Fig. 7H, I). Moreover, based on the quantification data from IHC staining, patients with USP7^high^TAZ^high^ had the worst survival as compared to other subgroups (Fig. 7J). Complementarily, correlation analyses of USP7 expression and Hippo-YAP/TAZ cancer signatures revealed that its mRNA abundance was significantly correlated with three previously reported Hippo-YAP/TAZ signatures using TCGA-HNSC dataset (*p* < 0.05, Supplementary Fig. 13A) [9, 34, 45]. In addition, bioinformatics analyses of transcriptomic datasets from Cancer Cell Line Encyclopedia database revealed significantly positive associations between USP7 expression and CYR61/CCN1 (*R* = 0.4498, *p* < 0.0111), CTGF/CCN2 (*R* = 0.3141, *p* < 0.0426) in 31 HNSCC cell lines (Fig. 7E and Supplementary Fig. 13B). To further unravel the biological significance of USP7-TAZ axis in HNSCC, we extracted the gene candidates positively associated with USP7 or TAZ in TCGA-HNSC samples (Spearman’s correlation >0.3) and found 440 overlapped genes which were further subjected to GO and KEGG analyses. As shown in Supplementary Fig. 14, these USP7/TAZ positively associated genes were significantly enriched in cancer-related epigenetic regulation and protein modifications. Taken together, these data provided ample evidence that USP7-TAZ axis served as key prognostic biomarkers and potential therapeutic targets in HNSCC.

## Discussion

The last decades have witnessed tremendous progress in elucidating key roles of Hippo-TAZ/YAP signaling across a myriad of physiopathological events especially in human cancer [6, 7, 33]. It has increasingly appreciated that Hippo core components and downstream effectors TAZ/YAP have been under tight regulations ranging from DNA variation to various post-translational modifications, which largely dictate the biological outcomes of this signaling in diverse contexts [7, 33]. Here, we identify USP7 as a novel regulator that physically interacts, functionally deubiquitinates and stabilizes TAZ, and define oncogenic USP7-TAZ axis driving HNSCC progression.

Mounting evidence has revealed aberrant overexpression and tumorigenic functions of TAZ across multiple cancers which are, to a great extent, governed by its protein abundance and cytoplasmic-nuclear distribution [36, 43]. Given the essential roles of ubiquitination and deubiquitination in protein hemostasis, pioneer works have revealed that TAZ ubiquitination and proteasomal degradation is predominantly mediated by β-TRCP [12, 14]. However, the antagonistic deubiquitinating enzymes for TAZ protein stability in cancer remain underexplored. Here we exploited unbiased DUBs screen and gain-/loss-of-function experimental approaches and unraveled USP7 as an essential DUB for TAZ protein stability in HNSCC. Recent reports have documented that TAZ can be directly deubiquitinated and stabilized by USP1 [46], USP10 [15], OTUB2 [16] and JOSD2 [47] in hepatocellular carcinoma, breast cancer and cholangiocarcinoma. Additionally, TAZ protein abundance was also indirectly modulated by other deubiquitinating enzymes such as USP9X [48] and YOD1 [49] via regulating Hippo signaling. However, under our experimental settings, we failed to reveal these reported DUB enzymes for modulating TAZ stability in HNSCC. We reasoned that this discrepancy might be attributed to diverse genetic background and etiologies among different cancers as well as experimental conditions. For example, poly-SUMOylated OUTB2 induced by EGF/KARS was required for interaction between OTUB2 and YAP/TAZ and subsequently YAP/TAZ stabilization by OTUB2 deubiquitination [16]. Recently, Zhou et al. have documented essential clues to support the notion that TAZ is a potential substrate for USP7, although their work and findings mainly focused on YAP deubiquitination by USP7 [50]. Moreover, previous results have shown that besides TAZ identified here, UPS7 have found to deubiquitinate multiple substrates intricately involved in cancer development such as p53/MDM2 [22, 23], HIF-1α [25], PTEN [51] and N-Myc [24]. This is in line with the fact that one protein can be substrate for multiple DUBs and one DUB can modulate several protein substrates under diverse biological contexts or experimental circumstances, as exemplified by PTEN and USP7, respectively [21, 38, 52]. We reasoned that USP7 might be a context-specific DUB for TAZ in HNSCC, which warranted further experimental validations.

Direct binding and interaction between DUBs and their substrates are required for deubiquitination. The D304/E308 residues in the central region of USP7 protein are critical for USP7-ubiquitin binding and substrate deubiquitination [30]. Our results derived from cellular immunofluorescence and subcellular fraction assays revealed that deubiquitination of TAZ by USP7 occurred mainly in nucleus, which agreed with the preferential nuclear localization of USP7 and activated TAZ [36, 53]. Furthermore, we mapped the potential binding regions between USP7 and TAZ protein and identified N-terminal region of USP7 and multiple regions of TAZ responsible for their interaction. Previous reports have unveiled N-terminal or C-terminal region of USP7 is required for substrates binding and interacting like Yki [50] and GATA1 [54], whereas both regions are needed for USP7 and p53 interaction [55]. Interestingly, USP7 substrates usually harbor multiple binding sites for USP7 as exemplified by 2 and 4 binding sites in p53 [22] and Gli [56], respectively. Our findings and the abovementioned results suggest a relatively common model for USP7 in binding via its N/C-terminal and subsequently deubiquitinating its substrates by central catalytic region [29, 57].

It has been well established that DUBs selectively recognize and remove polyubiquitin chains at diverse lysine sites of protein substrates and subsequently modulate their location, stabilities, abundance and functional activities [58]. Previous reports have pinpointed that K48- and K63-polyubiquitin are two best-characterized ubiquitin linkages cleaved by USP7 to promote stabilities or trafficking of specific substrates, respectively [29, 37]. Our results indicated that TAZ stabilization by USP7 relied on its DUB activity but independent of canonical Hippo cascade. Consistent with previous reports, USP7 preferentially cleaved K48-polyubiquitin chain but not K63-polyubiquitin chain of TAZ protein and subsequently prevented its proteasomal degradation.

The antagonistic actions between ubiquitin ligases and DUBs are responsible for protein homeostasis as exemplified that USP13-FBXL14 regulated reversible ubiquitination of c-Myc in glioblastoma stem cells [59], and USP15-SMURF2 cooperatively modulated TGF-β receptor in glioblastoma [60]. Indeed, similar antagonism between USP7 and β-TRCP on REST and β-catenin had been reported [61, 62]. Our results suggest that a steady level of TAZ protein might be under tight control by USP7 and β-TRCP through ubiquitination and deubiquitination processes. This notion was in part supported by similar findings that USP7 blocked β-TRCP-mediated YAP ubiquitination in a DUB-dependent manner [50]. However, given the preferential locations of USP7 and β-TRCP in nucleus or cytoplasm, we speculate that some unknown nuclear E3 ligases might exist for TAZ ubiquitination. Recently, Jiang Jin et al. have identified a nodular E3 ubiquitin ligase complex CRL4^DCAF12^ for YAP/TAZ ubiquitination and degradation in nucleus [42]. However, our results failed to support this finding. Furthermore, we reasoned that this balance was shifted toward reduced ubiquitination and increased deubiquitination of TAZ ultimately resulting in TAZ upregulation, which was presumably due to overexpressed USP7 and downregulated β-TRCP in HNSCC. This interesting idea warrants further experimental validations.

The biological functions of TAZ are largely dictated by its cytoplasmic-nuclear shuttling and subcellular location. TAZ relocates from nucleus to cytoplasm via 14-3-3 zeta- and CRM1-dependent pathways [44, 63]. Notably, a line of evidence has revealed that substrates deubiquitination by USP7 might affect their nuclear-cytoplasmic trafficking and in turn modulated their functional output [35, 64]. In line with this, our results revealed that pharmacological inhibition of USP7 by GNE6640 reduced ubiquitin chain removal on nuclear TAZ protein and in turn triggered TAZ nuclear export to cytoplasm. Moreover, stabilized TAZ by USP7 markedly increased its binding with TEADs in nucleus. These results are well consistent with the notion that the transcriptional factors TEADs bind with activated TAZ to form a complex which facilitates TAZ nuclear retention and drives its transcriptional output [36]. Moreover, recent work has pinpointed that TEADs bind with and masked nuclear export signal on TAZ (60-125aa, C-terminal) and impair its nuclear efflux, thus increasing its nuclear retention [44]. In aggregate, our results strongly suggest that following deubiquitination and stabilization by USP7, TAZ was retained in nucleus to sustain its transcriptional outputs to drive malignant behaviors in HNSCC.

Previous studies have documented that USP7 may play contradictory roles, pro-tumorigenic or anti-neoplastic, across diverse cancer contexts. Although USP7 acts as a tumor suppressor by stabilizing p53 [22, 23], it might serve as a putative oncogene as evidenced by overexpression, positive associations with malignant clinicopathological features as well as unfavorable survival in multiple human cancers [24, 26]. Our results agree with these findings and reiterate the upregulation, pro-tumorigenic functions and prognostic significance of USP7 in HNSCC. In supporting this, USP7 deubiquitinates and stabilizes well-established oncogenes such as HIF-1α and N-Myc to facilitate tumor progression [24, 25]. Our in vitro rescue experiments revealed that TAZ functioned as a novel downstream target of USP7 to promote HNSCC progression as evidenced that reintroduction TAZ largely attenuated the biological effects of USP7 knockdown. Complementarily, strong positive correlations between USP7 and TAZ protein and prominent overlapping of USP7-/TAZ-associated genes in HNSCC clinical samples offer support to the notion that TAZ likely serves as one of USP7 pro-tumorigenic mediators in HNSCC. Of course, we cannot rule out the possibility that other uncharacterized downstream mediators beyond TAZ exist in HNSCC, which warrants further explorations.

**Fig. 8:**
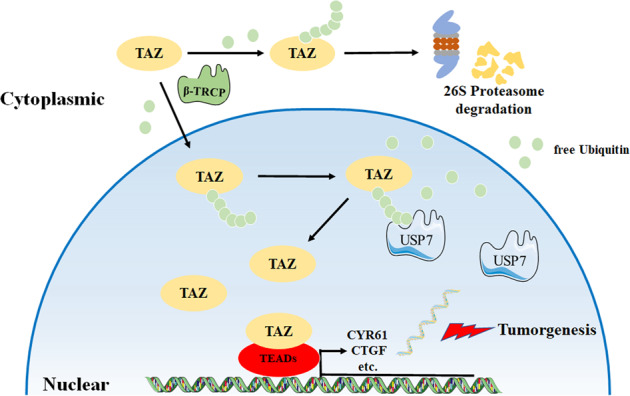
A proposed model of TAZ deubiquitination and stabilization by USP7 in HNSCC. TAZ protein is under tight control by USP7 and β-TRCP through ubiquitination and deubiquitination processes. Upon TAZ translocates into nucleus, USP7 interacts with, deubiquitinates and stabilizes TAZ as well as facilitates its nuclear retention to activate transcription of downstream targets to drive HNSCC progression.

Given the great translational potentials of DUB as druggable targets, selective USP7 chemical inhibitors represent one of most extensively investigated compounds which have shown promising results in preclinical models [27, 65]. In addition, given its prominent role during tumorigenesis, TAZ still remains undruggable. Our findings reinforced that USP7 was a novel viable therapeutic target for HNSCC as genetic depletion or pharmacological inhibition of USP7 resulted in reduced tumor growth in vivo likely in part via TAZ inhibition. To further consolidate the clinical significance of USP7-TAZ axis, our data revealed that concomitantly upregulated USP7 and TAZ robustly stratified patients into subgroups with diverse survival rates. Collectively, we conclude that USP7-TAZ axis plays putative oncogenic roles in HNSCC and can be exploited as novel prognostic biomarkers and therapeutic targets with translational promises (Fig. 8).

In conclusion, we identify USP7 as a novel bona fide DUB for TAZ protein stability and define oncogenic USP7-TAZ axis to facilitate HNSCC progression. Therapeutically targeting this axis represents a viable novel therapeutic strategy for HNSCC. Our findings offer insights into aberrant TAZ overexpression underlying tumorigenesis and may advance the development of therapeutic approaches to disrupt the USP7-TAZ axis in HNSCC.

## Supplementary information

Supplementary Figure 1

Supplementary Figure 2

Supplementary Figure 3

Supplementary Figure 4

Supplementary Figure 5

Supplementary Figure 6

Supplementary Figure 7

Supplementary Figure 8

Supplementary Figure 9

Supplementary Figure 10

Supplementary Figure 11

Supplementary Figure 12

Supplementary Figure 13

Supplementary Figure 14

Supplementary Figure legends

Supplementary Table 1-6

Original Western Blots

Author Contribution Statement

Reproducibility checklist

## Data Availability

The materials and datasets used are available from corresponding authors on reasonable request.

## Code availability

All code runs performed by related R packages rely on R 4.1.1. The gene sets signature annotation was downloaded from MsigDB (http://www.gsea-msigdb.org/gsea/msigdb/index.jsp, Hallmark: h.all.v7.2.entrez.gmt; GO: c5.go.v7.2.entrez.gmt; KEGG: c2.cp.kegg.v7.2.entrez.gmt) [66].
