# Supplementary figures and images for: The deubiquitinase USP7 promotes HNSCC progression via deubiquitinating and stabilizing TAZ
Source: Cell Death Dis. 2022 Aug 5;13(8):677. doi: 10.1038/s41419-022-05113-z (PMC9356134; doi:10.1038/s41419-022-05113-z)

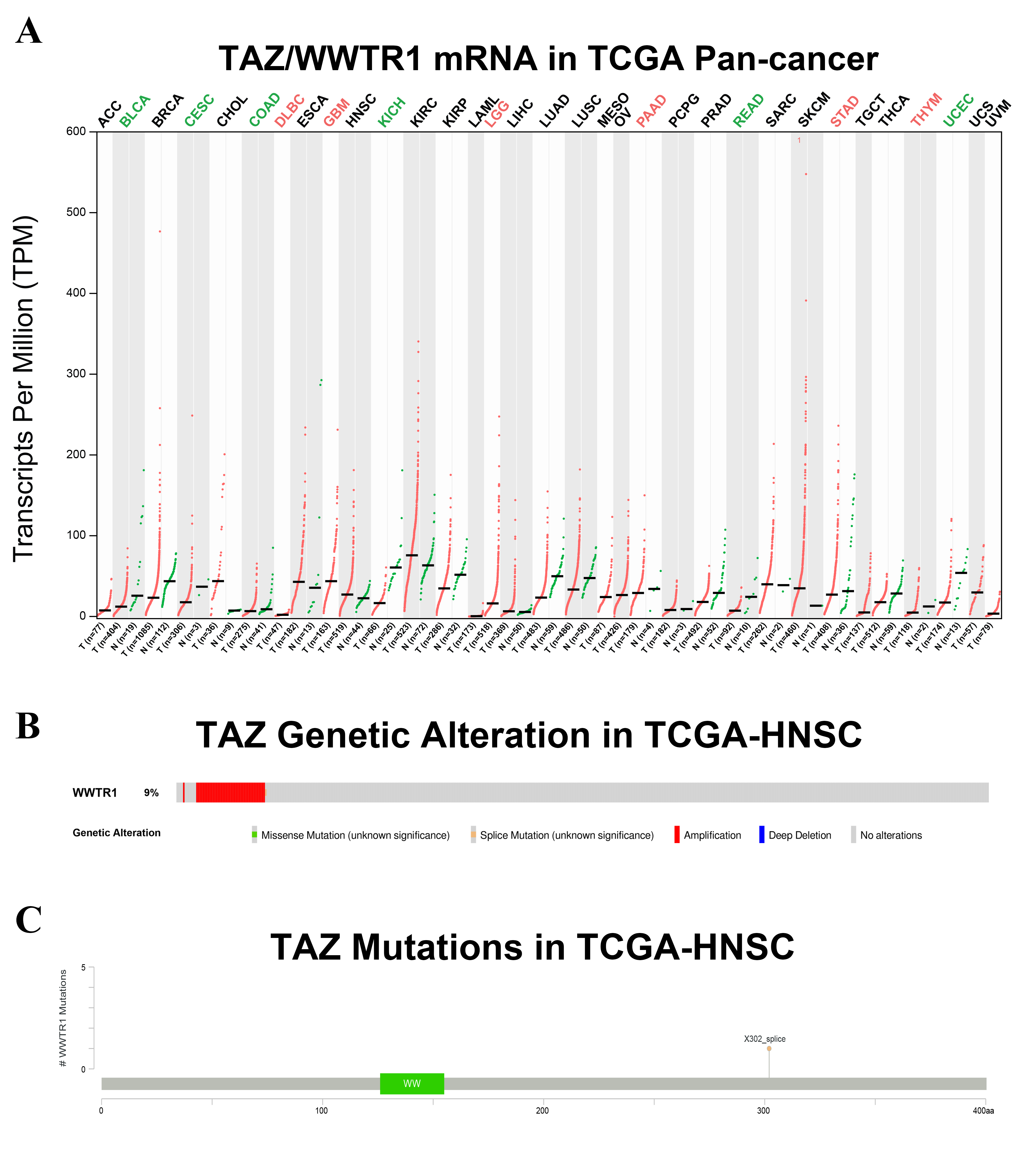

Supplement: Supplementary file 1 — Supplementary Figure 1 [file 41419_2022_5113_MOESM1_ESM.tif]

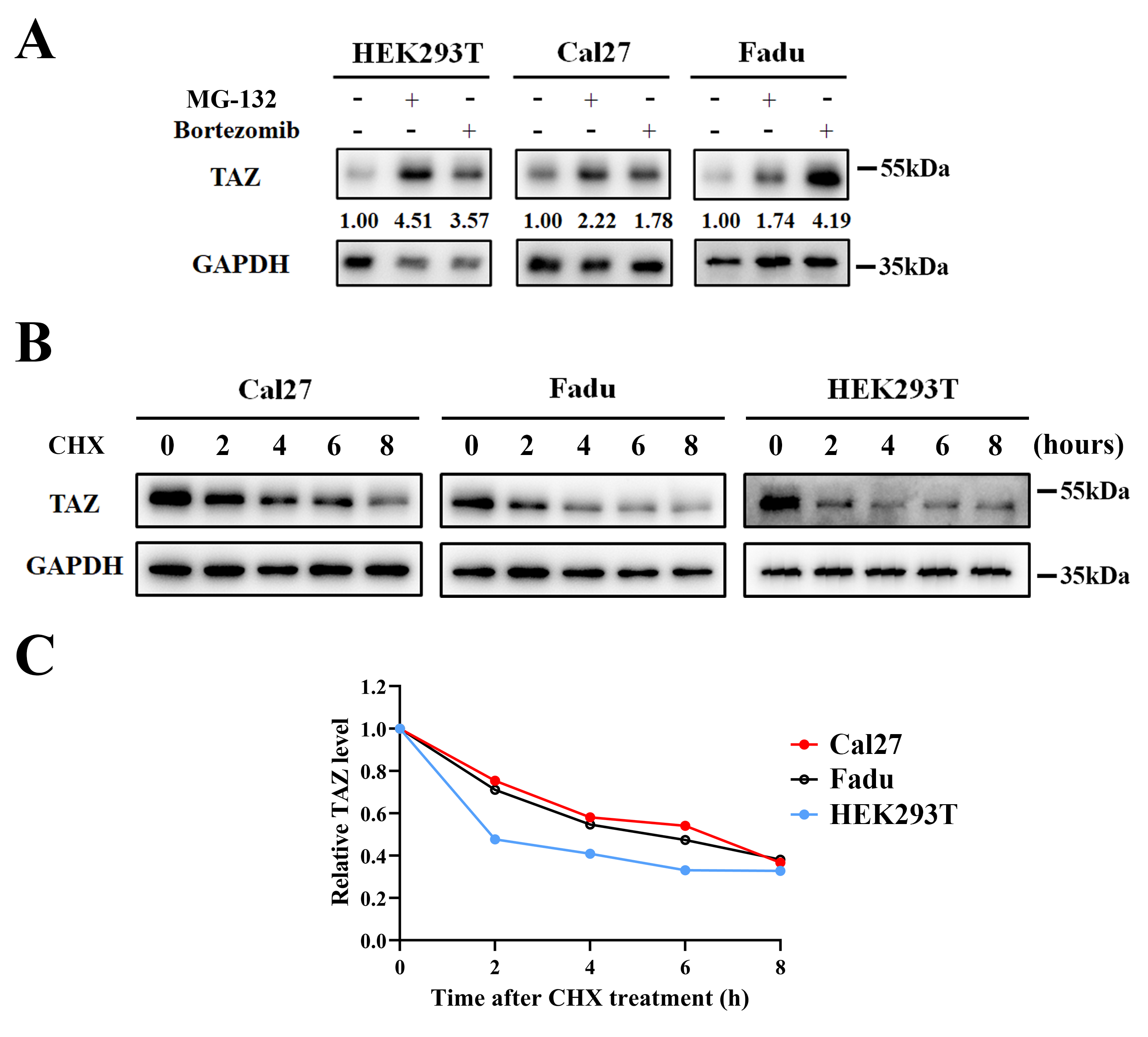

Supplement: Supplementary file 2 — Supplementary Figure 2 [file 41419_2022_5113_MOESM2_ESM.tif]

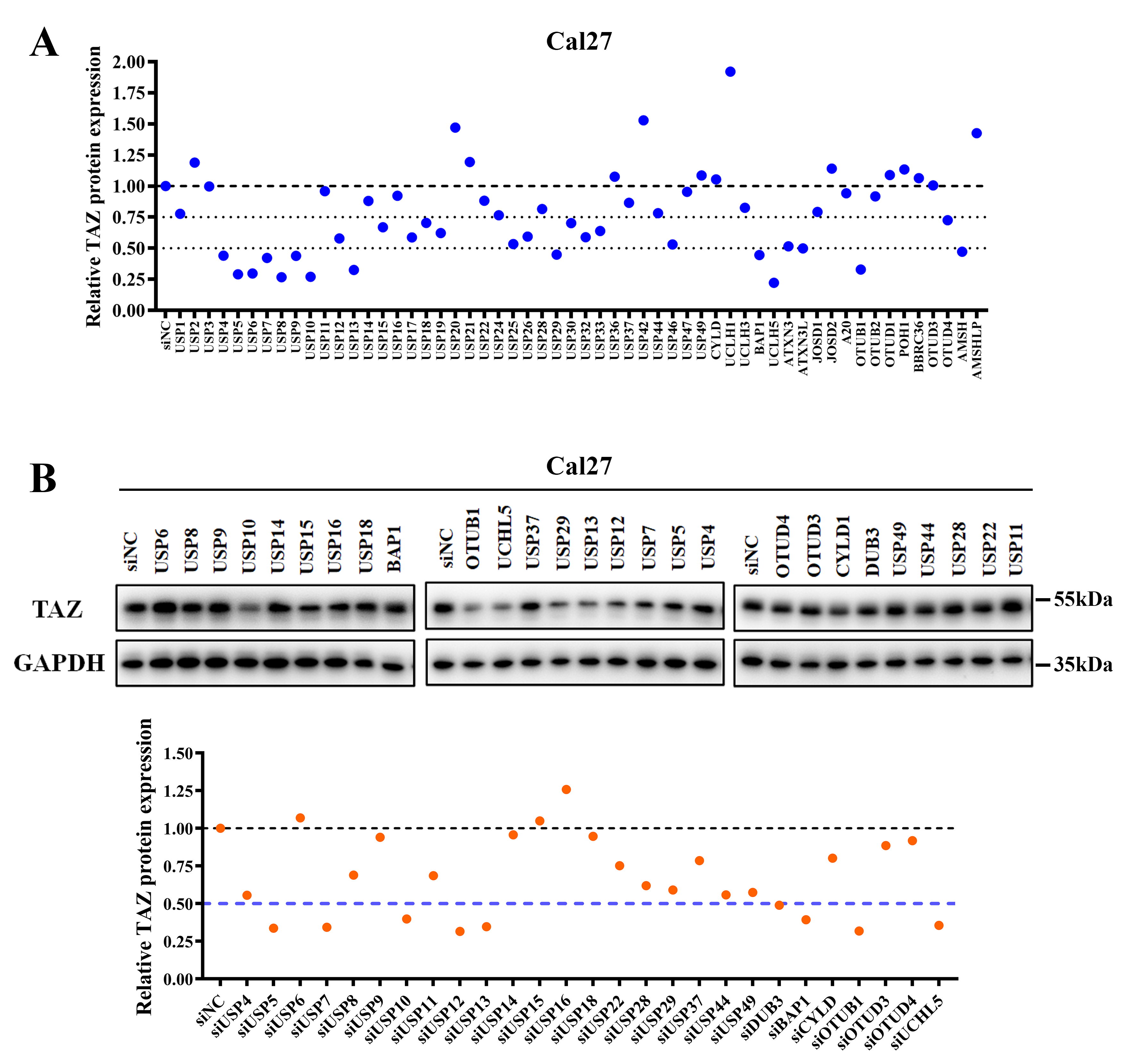

Supplement: Supplementary file 3 — Supplementary Figure 3 [file 41419_2022_5113_MOESM3_ESM.tif]

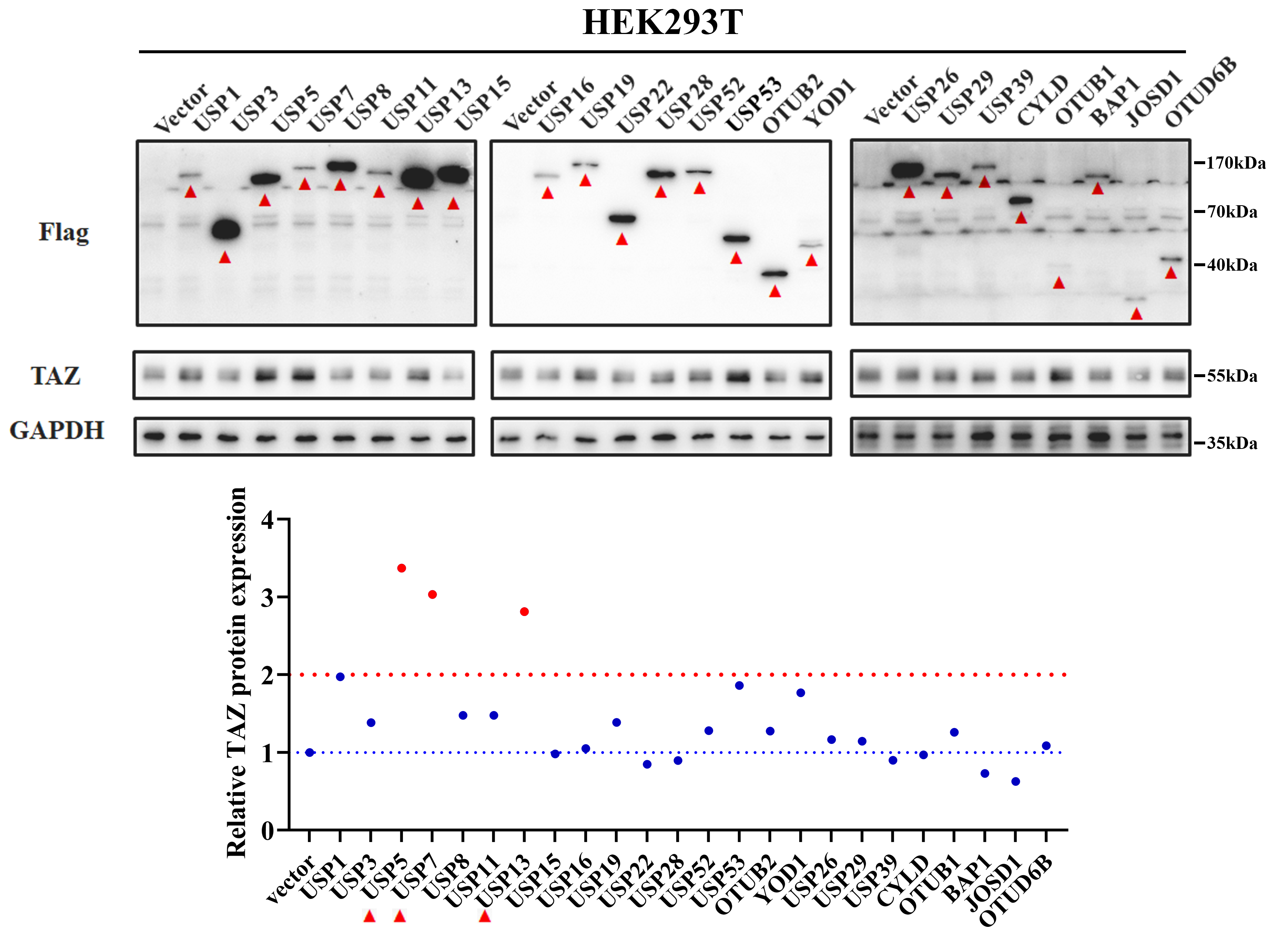

Supplement: Supplementary file 4 — Supplementary Figure 4 [file 41419_2022_5113_MOESM4_ESM.tif]

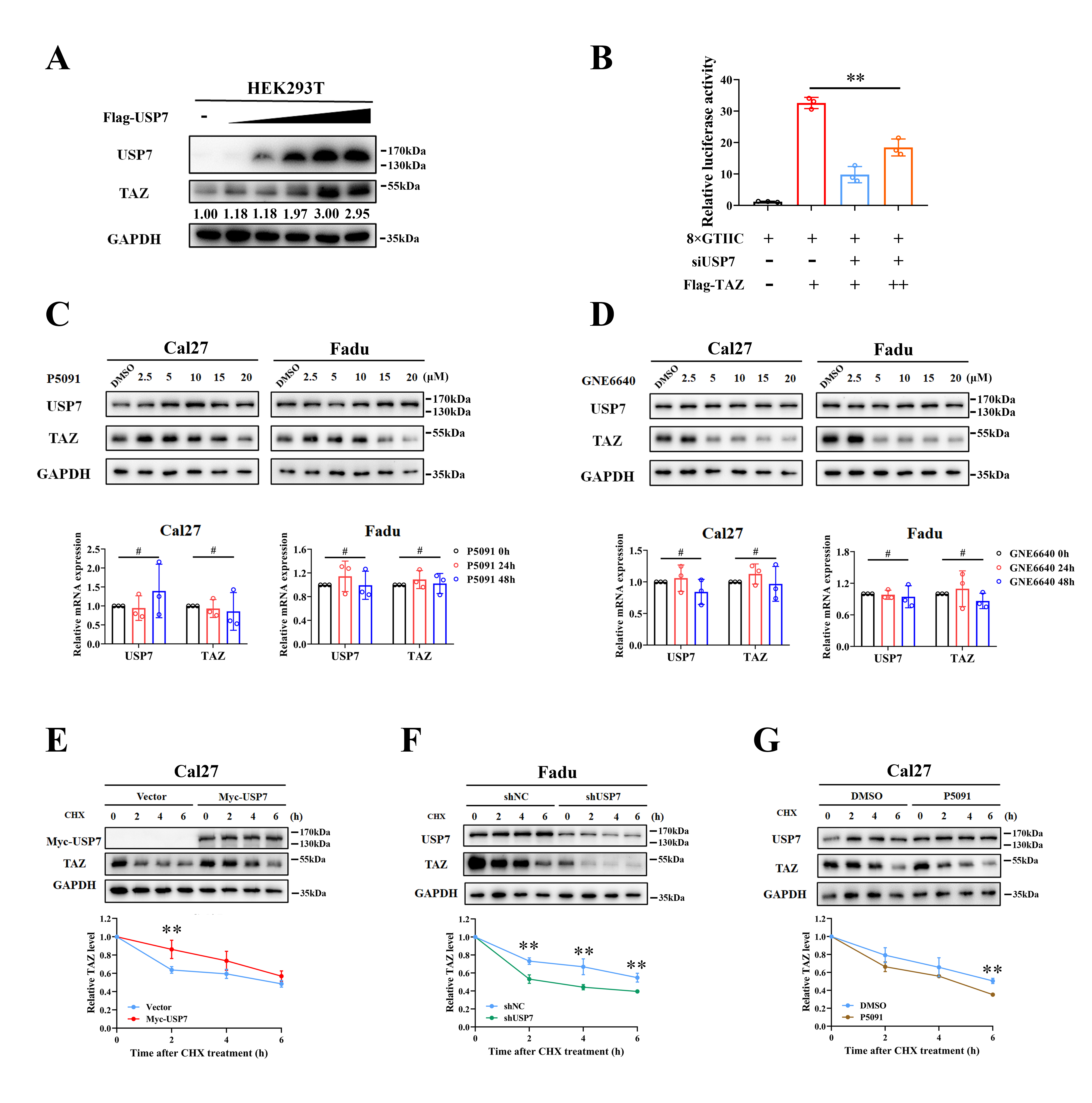

Supplement: Supplementary file 5 — Supplementary Figure 5 [file 41419_2022_5113_MOESM5_ESM.tif]

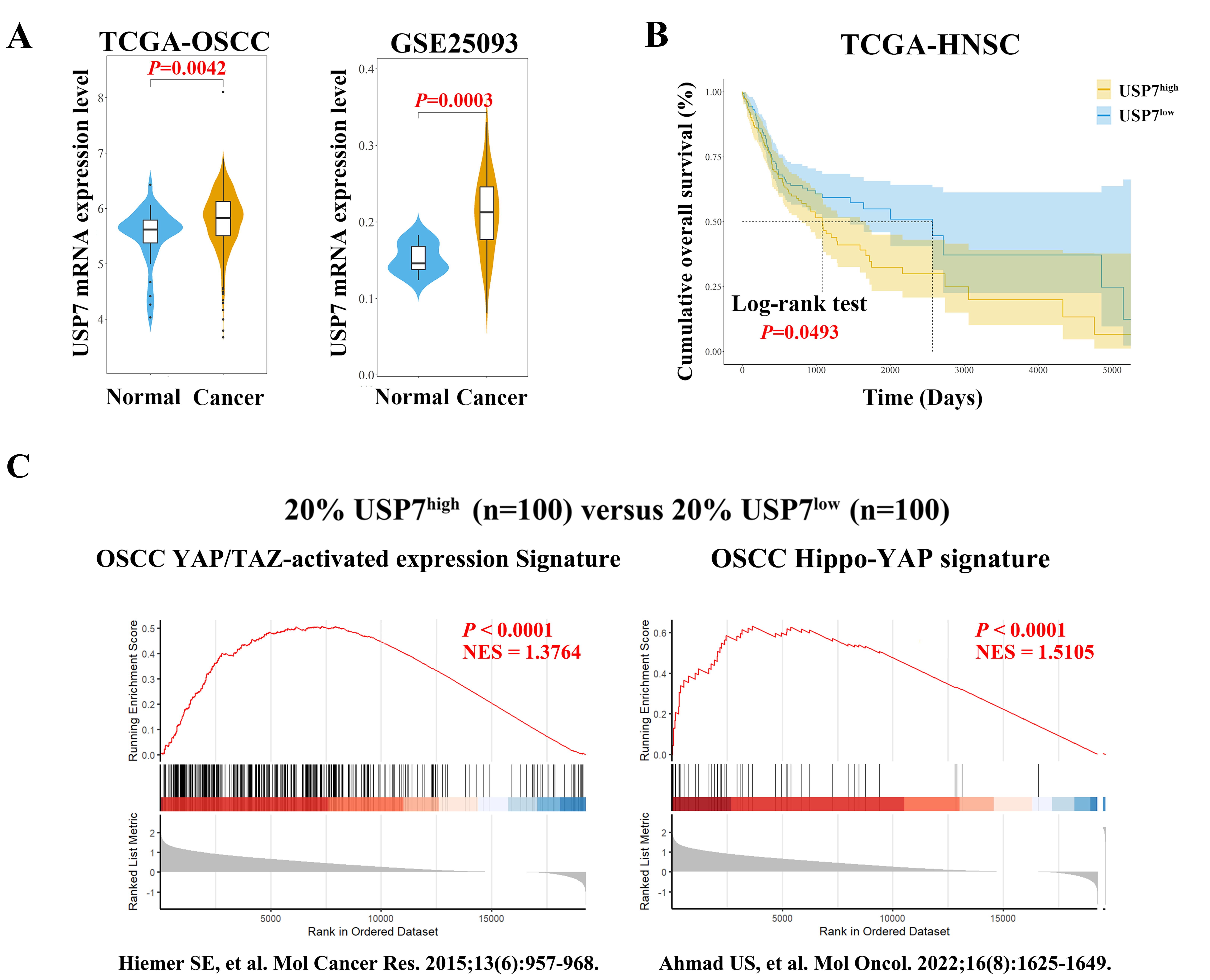

Supplement: Supplementary file 6 — Supplementary Figure 6 [file 41419_2022_5113_MOESM6_ESM.tif]

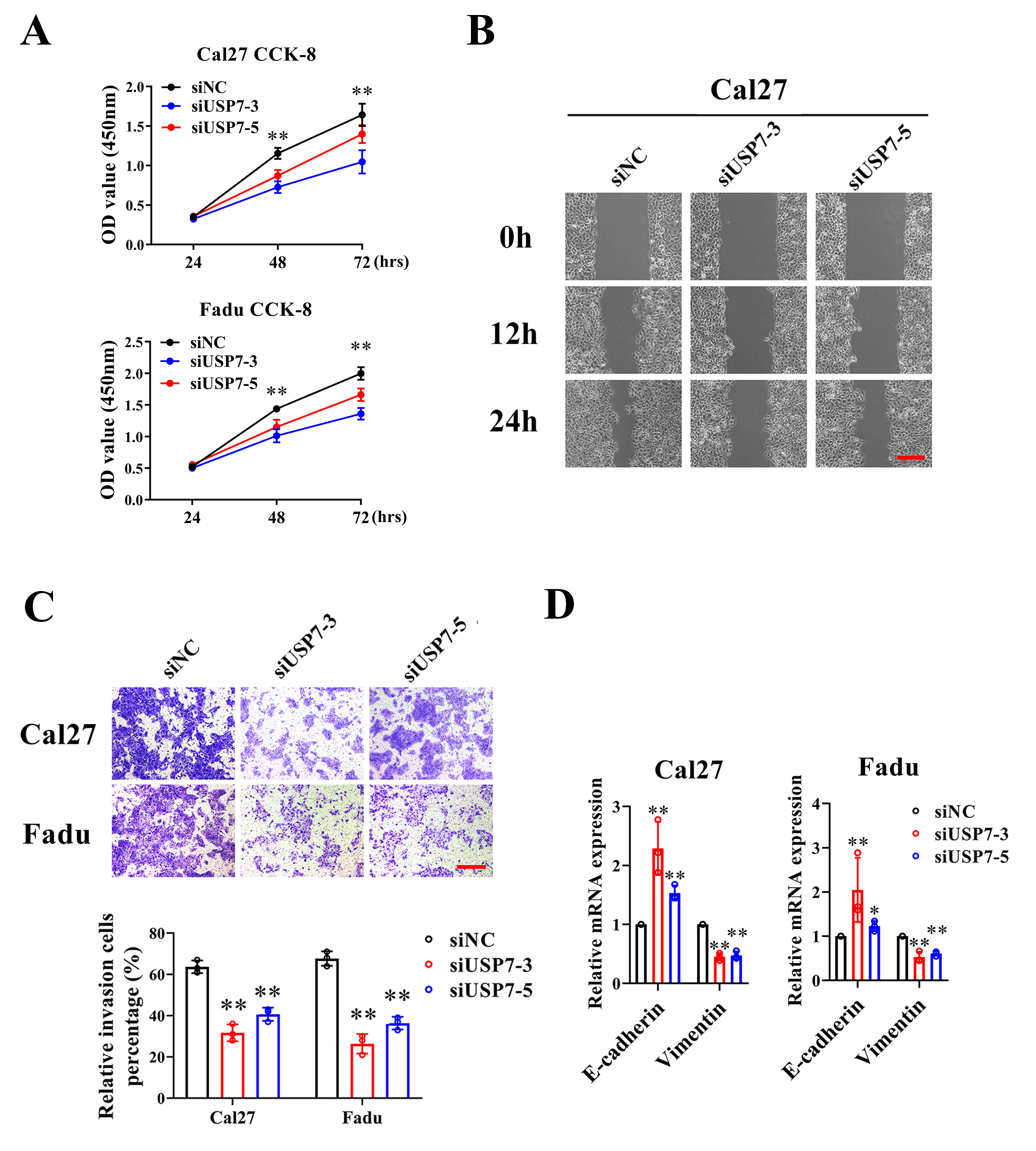

Supplement: Supplementary file 7 — Supplementary Figure 7 [file 41419_2022_5113_MOESM7_ESM.tif]

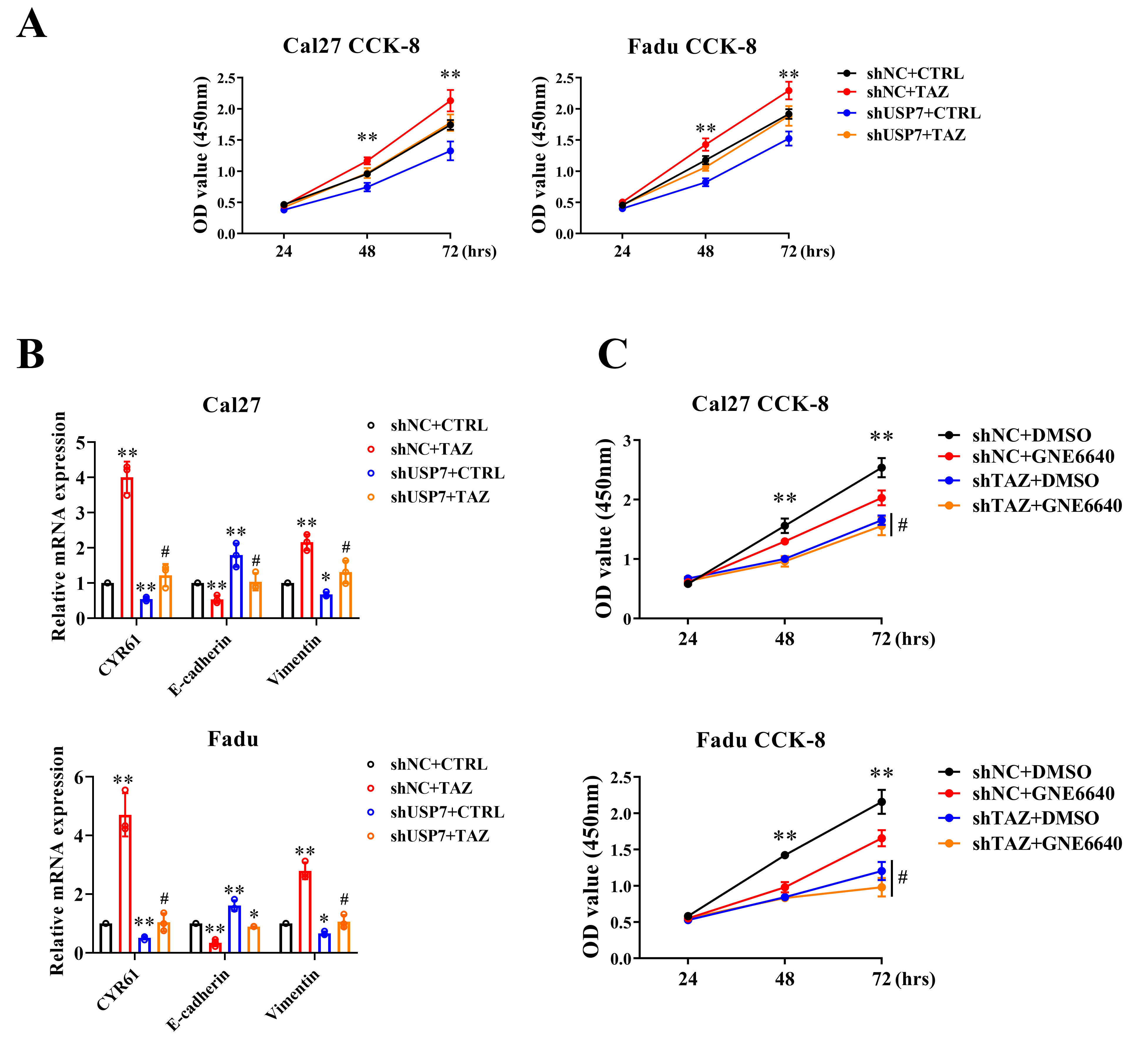

Supplement: Supplementary file 8 — Supplementary Figure 8 [file 41419_2022_5113_MOESM8_ESM.tif]

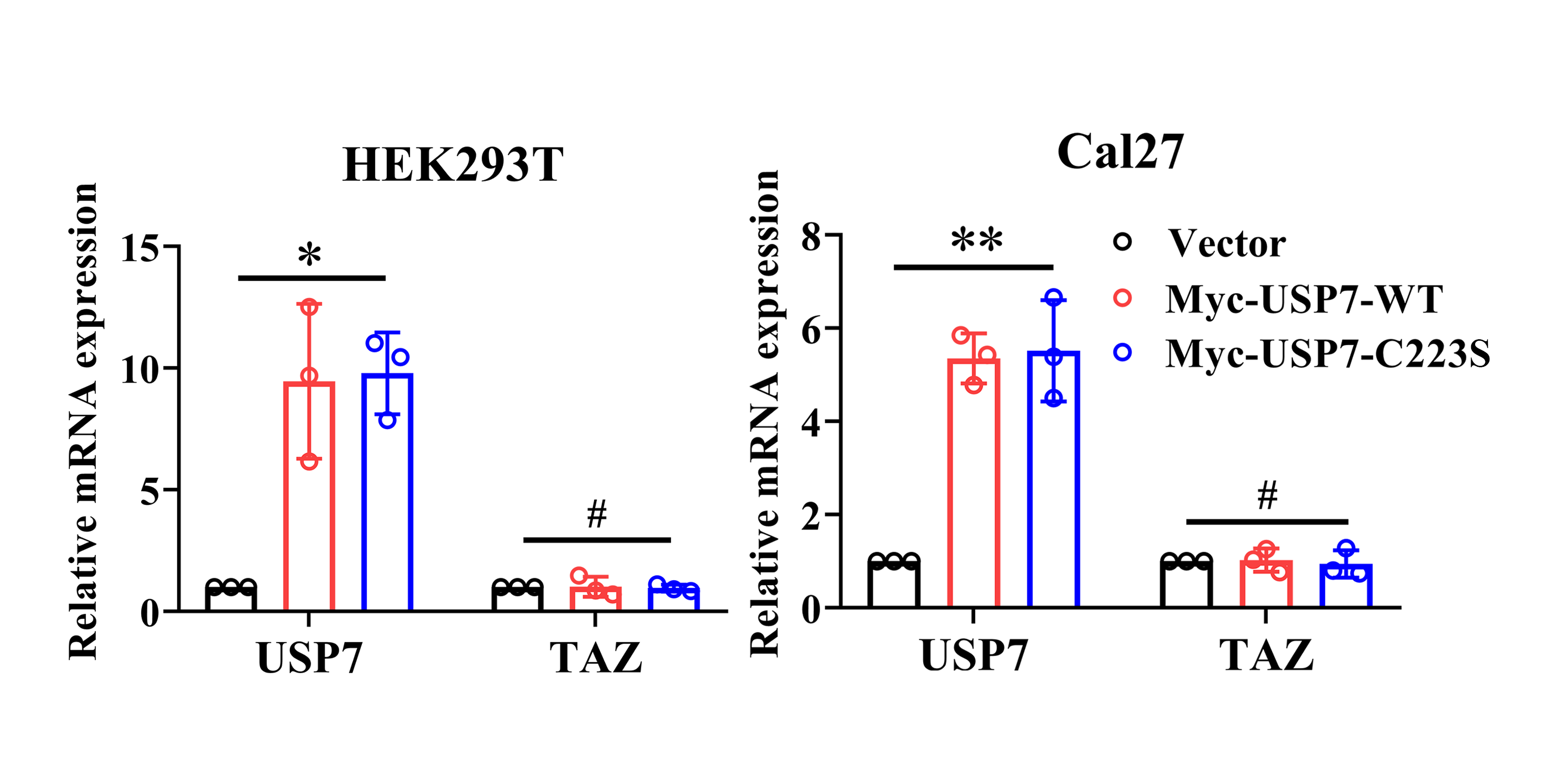

Supplement: Supplementary file 9 — Supplementary Figure 9 [file 41419_2022_5113_MOESM9_ESM.tif]

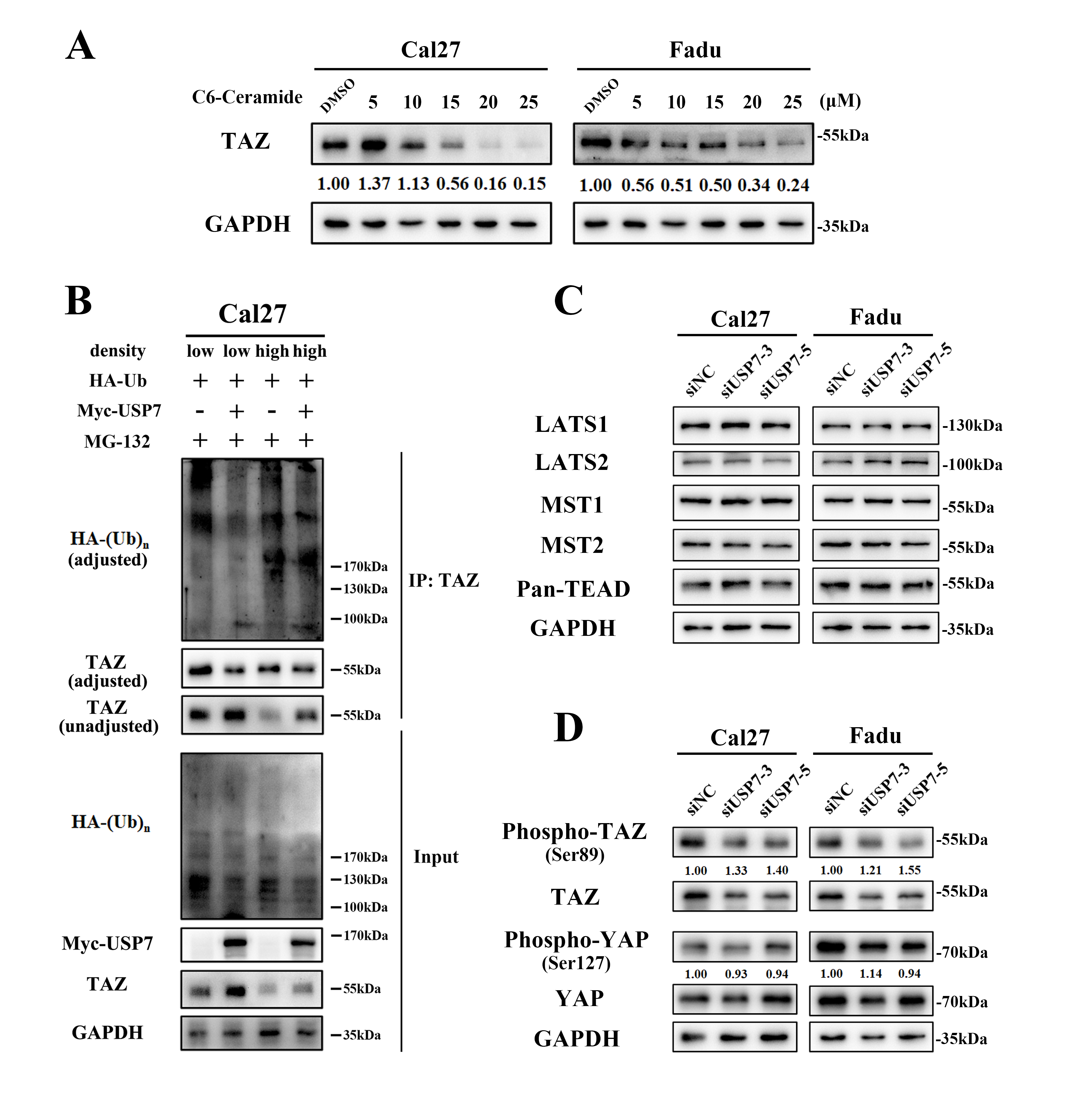

Supplement: Supplementary file 10 — Supplementary Figure 10 [file 41419_2022_5113_MOESM10_ESM.tif]

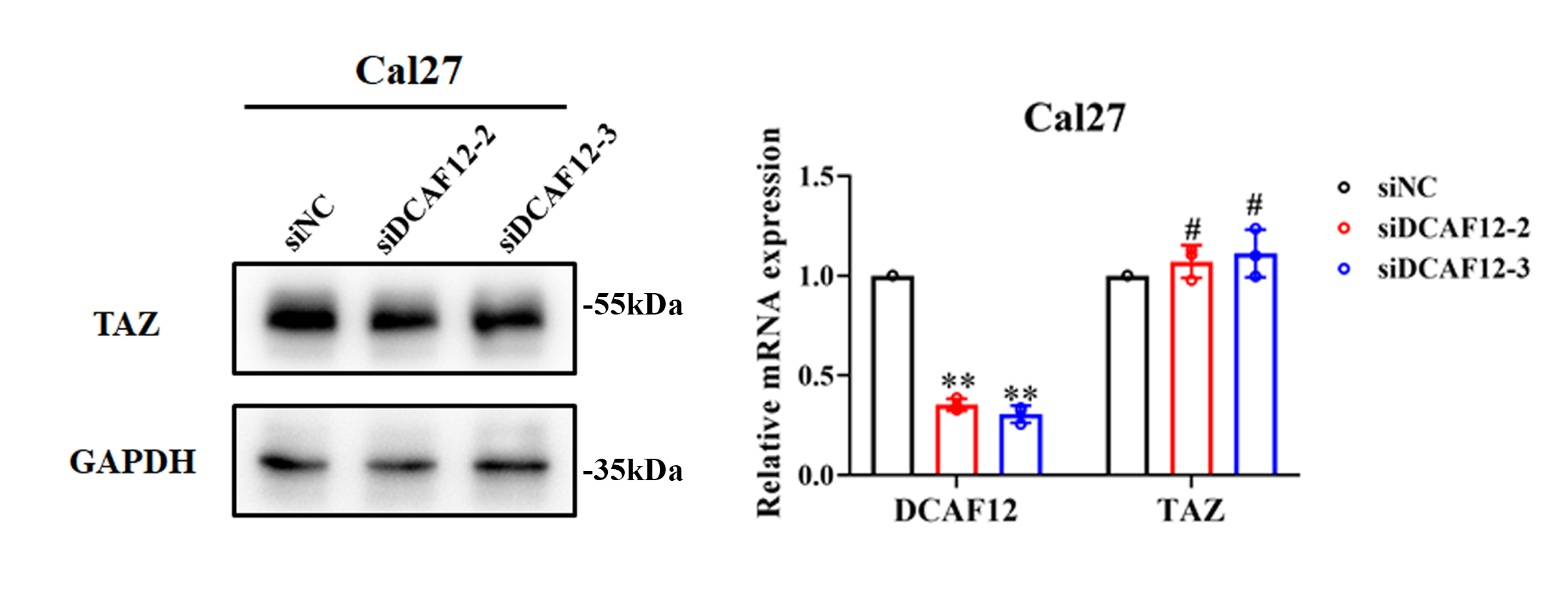

Supplement: Supplementary file 11 — Supplementary Figure 11 [file 41419_2022_5113_MOESM11_ESM.tif]

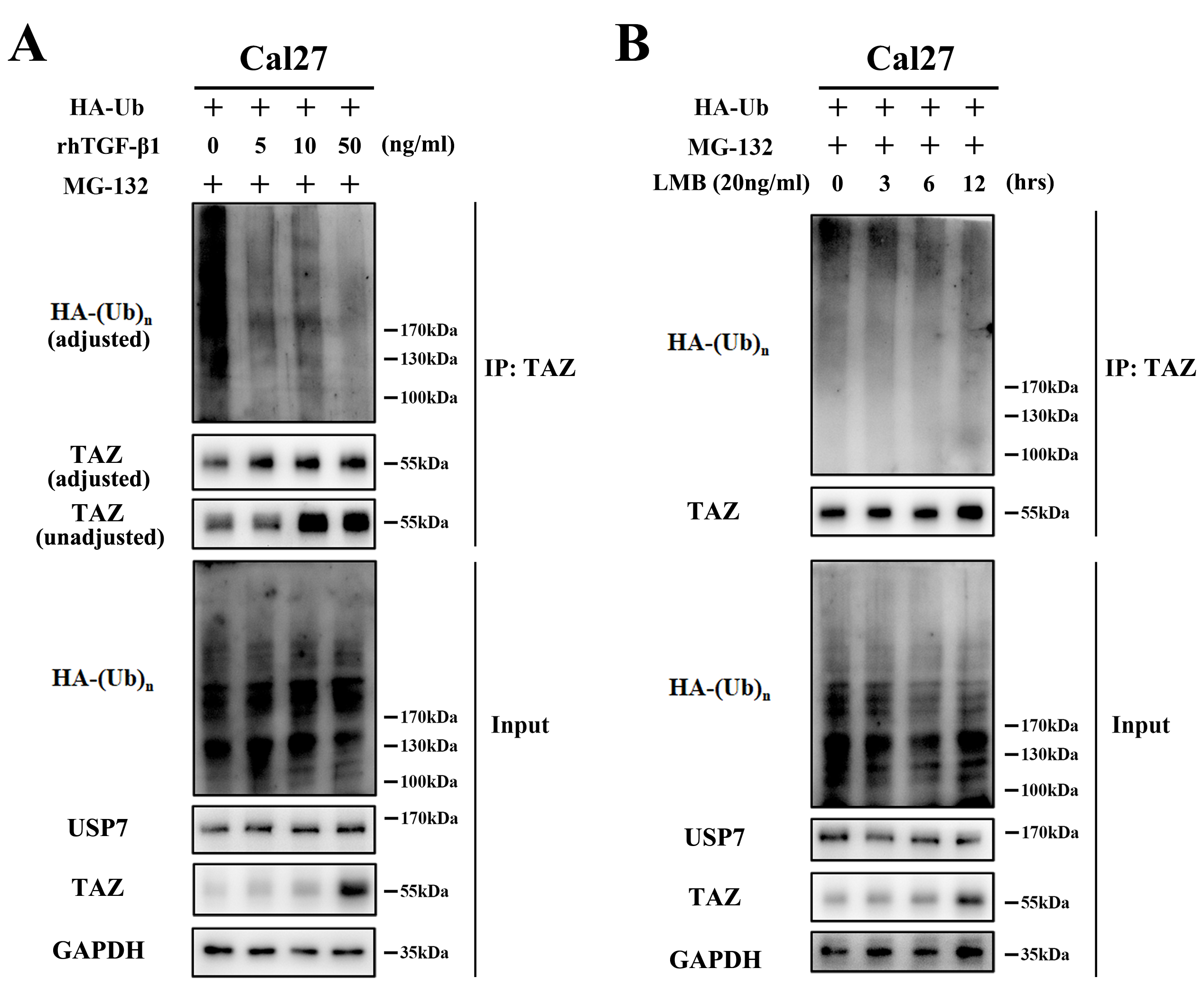

Supplement: Supplementary file 12 — Supplementary Figure 12 [file 41419_2022_5113_MOESM12_ESM.tif]

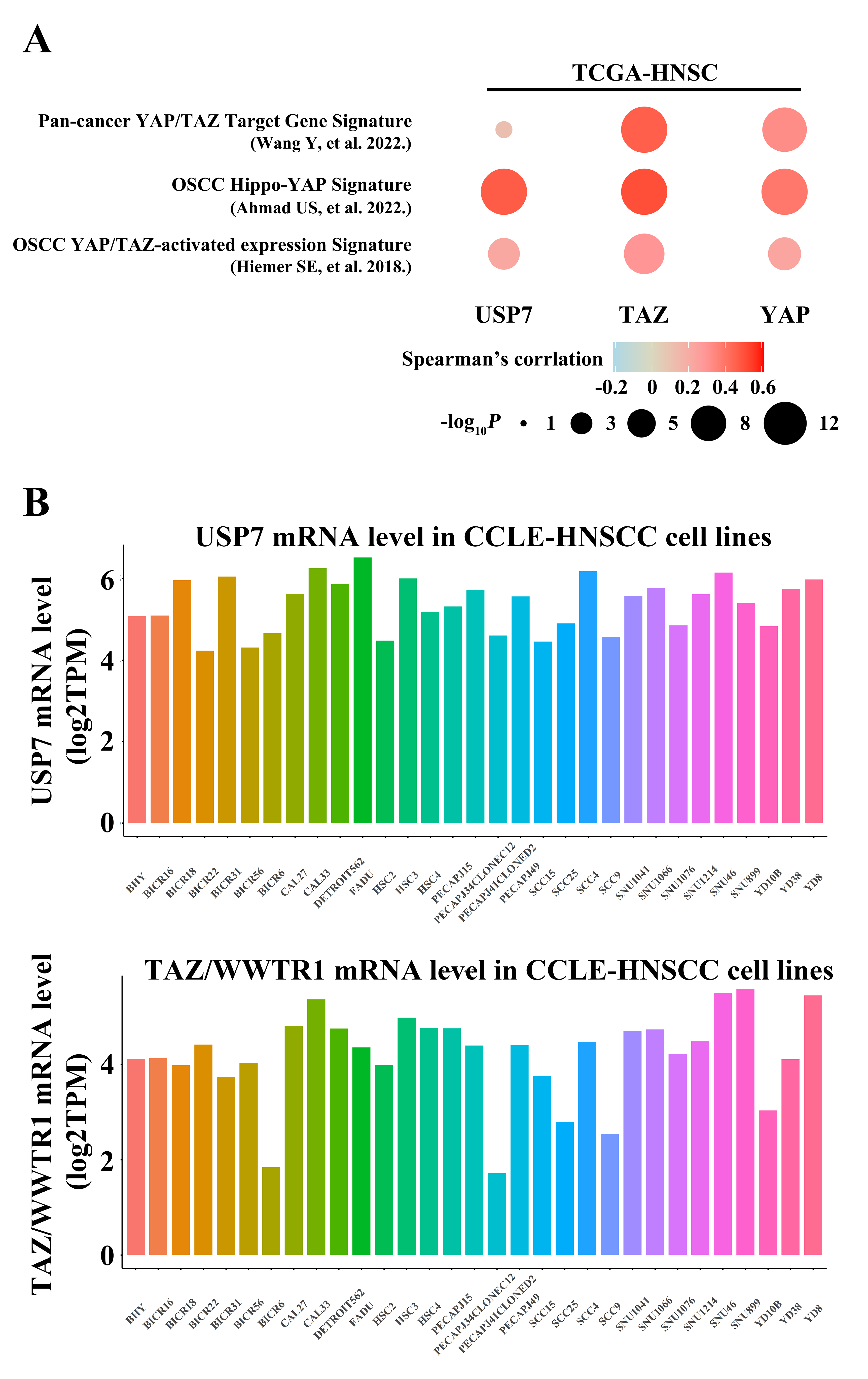

Supplement: Supplementary file 13 — Supplementary Figure 13 [file 41419_2022_5113_MOESM13_ESM.tif]

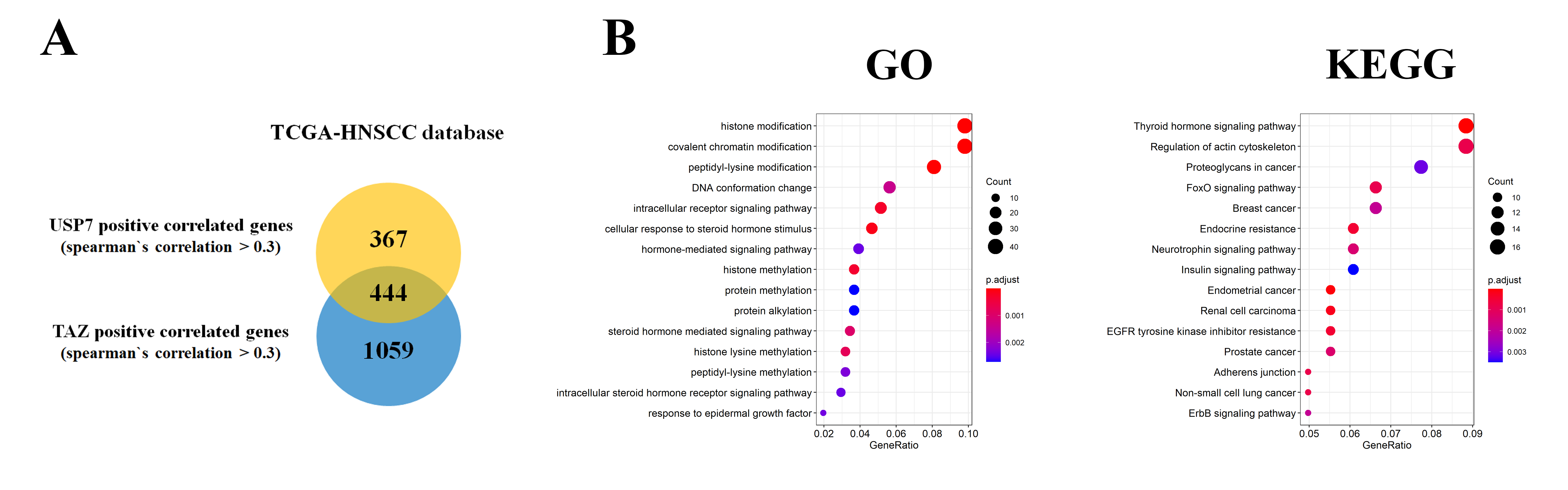

Supplement: Supplementary file 14 — Supplementary Figure 14 [file 41419_2022_5113_MOESM14_ESM.tif]
