# Supplementary material for: The deubiquitinase USP7 promotes HNSCC progression via deubiquitinating and stabilizing TAZ
Source: Cell Death Dis. 2022 Aug 5;13(8):677. doi: 10.1038/s41419-022-05113-z (PMC9356134; doi:10.1038/s41419-022-05113-z)
Supplement: Supplementary file 15 — Supplementary Figure legends [file 41419_2022_5113_MOESM15_ESM.docx]

**Supplementary Figure legends**

**Supplementary Figure 1. Pan-cancer TAZ mRNA expression.**

**A.** The mRNA expression of TAZ/WWTR1 across various human cancers using datasets extracted from online cBioPortal database.

**B.** Genetic alterations of TAZ in TCGA-HNSC samples. A total of 496 TCGA-HNSC samples were included and analyzed.

**C.** Diagram of TAZ mutations in TCGA-HNSC samples.

**Supplementary Figure 2.** **TAZ protein is regulated by** **proteasome-mediated degradation.**

**A.** The protein changes of TAZ were determined by western blot in HEK293T, Cal27 and Fadu cells treated with two proteasome inhibitors MG-132(10μM) or Bortezomib (50nM) for 8 hours.

**B.** The half-life of TAZ protein was measured in HEK293T, Cal27 and Fadu cells as gauged in the CHX chase assay.

Representative images were shown.

**Supplementary Figure 3. DUBs siRNA library screen.**

**A-B.** The protein changes of TAZ were determined by western blot in Cal27 cells transfected with individual siRNAs targeting selected DUBs. Quantification of the results was shown below.

**Supplementary Figure 4. DUBs cDNA library screen.**

The protein changes of TAZ were determined by western blot in HEK293T cells individually transfected with selected Flag-tagged DUBs cDNA plasmids, followed by immunoblot with antibodies against Flag, TAZ and GAPDH. Quantification of the results was shown below.

**Supplementary Figure 5. Pharmacological inhibition of USP7 facilitates TAZ protein decay.**

**A.** Endogenous TAZ protein expression was detected in HEK293 cells transfected with increased doses (0, 100, 500, 1000, 2000, 3000ng) of Flag-USP7 cDNA plasmids.

**B.** Luciferase activities on TEAD-binding site were measured in HEK293T cells transfected with siUSP7 and/or Flag-tagged TAZ cDNA plasmid via luciferase report assay.

**C-D.** Endogenous TAZ protein and mRNA were determined via western blot or qRT-PCR in Cal27 and Fadu cells treated with USP7 inhibitors P5091 (**C**) or GNE6640 (**D**), respectively.

**E-G.** The half-life of TAZ protein was detected by CHX assays when cells were treated with Myc-USP7 cDNA plasmid (**E**), shUSP7 (**F**) or P5091 (**G**). Quantification of the results was shown below.

Data were presented with representative images from 3 independent experiments.

**Supplementary Figure 6. USP7 was overexpressed in HNSCC samples and associated with** **Hippo-YAP/TAZ gene signatures and patient survival.**

**A.** USP7 mRNA level (log2-transformed) was significantly overexpressed in cancer samples derived from TCGA-OSCC and GSE25093 cohort as compared to non-tumor counterparts.

**B.** Kaplan-Meier plots indicated that patients stratified with high expression of USP7 (median-cutoff) had significantly lower overall survival rates relative to those with low expression USP7 (Log-rank test).

**C.** Enrichment plots of GSEA analyses showed that these differentially expressed genes between 20% USP7^high^ and 20% USP7^low^ samples were significantly enriched in two Hippo-YAP/TAZ cancer signatures.

**Supplementary Figure 7 (Related to Figure 2) USP7 silencing impaired tumor malignant phenotype in HNSCC cells**

**A.** The proliferative potentials of Cal27 and Fadu cells were significantly reduced upon USP7 knockdown as measured by CCK-8 assay.

**B-C.** Cell migration of Cal27 (**B**) and invasion of Cal27 and Fadu (**C**) were remarkably impaired after USP7 silencing. Data of crystal violet staining cells of each field were counted (**C**, lower panel). Scale bar: 100μm.

**D.** Selected markers associated with cell migration and invasion were markedly downregulated following siRNA-medicated USP7 knockdown.

Data were presented as mean ± SD from 3 independent experiments. Student`s *t* test or One-way ANOVA test. **P* < 0.05, ***P* < 0.01.

**Supplementary Figure 8.** **Enforced TAZ overexpression potently attenuated the phenotypic effects induced by USP7 knockdown.**

**A.** Cell viability and proliferation were measured in CCK-8 assays in cells treated with shUSP7 and/or TAZ cDNA plasmid.

**B.** The abundances of E-cadherin, N-cadherin and Vimentin were measured by qRT-PCR in cells treated with shUSP7 and/or TAZ cDNA plasmid.

**C.** CCK-8 assay showed that GNE6640 treatment has a minimum effect on TAZ knockdown cell lines.

Representative images were shown. Data were presented as mean ± SD from 3 independent experiments. Student`s *t* test or One-way ANOVA test. ^#^*P* ≥ 0.05, **P* < 0.05, ***P* < 0.01.

**Supplementary Figure 9** **(Related to Figure 4) USP7 overexpression undistributed TAZ mRNA level.**

Endogenous TAZ mRNA was detected by qRT-PCR in HEK293T cells transfected with empty vector, Myc-USP7-WT or Myc-USP7-C223S for 48 hours. Data were presented as mean ± SD from 3 independent experiments. ANOVA test. **P* < 0.05, ***P* < 0.01.

**Supplementary Figure 10 (Related to Figure 5) USP7 knockdown unaffected other Hippo core members expression.**

**A.** Endogenous TAZ protein was efficiently reduced by C6-Ceramid in a dosage-dependent manner in Cal27 and Fadu cells for 6h.

**B.** Cal27 cells at low or high densities were co-transfected with HA-Ub with/without Myc-USP7 and treated with MG-132 for 8 hours before cell collection. Exogenous TAZ ubiquitination was determined by immunoprecipitation by anti-TAZ antibody and immunoblotted with anti-HA antibody.

**C.** USP7 knockdown had minimal effects on Hippo core components (LATS1, LATS2, MST1, MST2 and TEAD) as determined by western blot in both Cal27 and Fadu cells.

**D.** USP7 knockdown had minimal effects on phosphorylated TAZ and its paralog YAP as detected by western blot in both Cal27 and Fadu cells. The phosphorylation ratio was qualified and given (phosphorylated/total)

Representative images of western blot were shown.

**Supplementary Figure 11. DCAF12 knockdown has marginal effects on TAZ protein abundance.**

The protein abundance of TAZ was measured by western blot in Cal27 cells transfected with two independent siRNA targeting DCAF12. Representative images were shown.

**Supplementary Figure 12. Measurements of endogenous TAZ ubiquitination following rhTGF-β1 or LMB treatment**

A. Cal27 cells were transfected with HA-Ub plasmid for 48h and then treated rhTGF-β1 (0, 5, 10, 50 ng/ml, 24h) and MG-132 (10μM, 8h) before cell harvest. The whole cellular lysates and subcellular fractions (cytoplasmic and nuclear) were subjected to immunoprecipitation by anti-TAZ antibody and immunoblotted with anti-HA antibody.

B. Following treatments with LMB (20ng/ml, 0,3,6,12h) and MG-132 (10μM, 8h) in Cal27 cells, the whole cellular lysates and subcellular fractions (cytoplasmic and nuclear) were subjected to immunoprecipitation by anti-TAZ antibody and immunoblotted with anti-HA antibody.

**Supplementary Figure 13. USP7 expression is positive correlated with Hippo-YAP/TAZ gene signatures in HNSCC.**

**A.** Heatmap shows the correlations of USP7/TAZ/YAP mRNA abundance with three previously reported Hippo-YAP/TAZ signature scores. Circle size represents statistical significance (-log_10_*P*); color represents correlations.

**B.** The mRNA expression of USP7 and TAZ (log_2_TPM) in HNSCC cell lines from CCLE dataset were shown with bar plot. Data were extracted from CCLE database. A total number of 31 HNSCC cell lines were included.

**Supplementary Figure 14. USP7/TAZ positively-correlated genes are significantly enriched in several cancer-related pathways**

**A-B.** A total number of 444 overlapped genes were identified from USP7 positively-correlated and TAZ positively-correlated genes extracted from TCGA-HNSC datasets. These genes were subjected to GO and KEGG analyses.
