# Supplementary material for: The deubiquitinase USP7 promotes HNSCC progression via deubiquitinating and stabilizing TAZ
Source: Cell Death Dis. 2022 Aug 5;13(8):677. doi: 10.1038/s41419-022-05113-z (PMC9356134; doi:10.1038/s41419-022-05113-z)
Supplement: Supplementary file 16 — Supplementary Table 1-6 [file 41419_2022_5113_MOESM16_ESM.docx]

**Supplementary Table 1: Chemical compounds and Recombinant Cytokine:**

| **Product** | **Source** | **Catalog number** |
| --- | --- | --- |
| MG-132 | Selleck | S2619 |
| Bortezomib (PS-341) | Selleck | S1013 |
| Cycloheximide (CHX) | Millipore | 5.08739 |
| P5091 | MedChemExpress | HY-15667 |
| GNE6640 | MedChemExpress | HY-112937 |
| C6-Ceramide (d18:1/6:0) | APExBIO | C5746 |
| Leptomycin B (LMB) | MedChemExpress | HY-16909 |
| Recombinant Human TGF-β1  (rhTGF-β1) | Peprotech | 100-21 |

**Supplementary Table 2: Human DUBs siRNA Library (57 DUBs)**

| **RNAi** | **Source** | **Sequence (5’-3’)** |
| --- | --- | --- |
| USP1-A | Genepharma | AACCCUAUGUAUGAAGGAUAU |
| USP1-B | Genepharma | TCGGCAATACTTGCTATCTTA |
| USP2-A | Genepharma | CAGATTGTGGTTACTGTTCTA |
| USP2-B | Genepharma | CAGGAGAATGGCACACTTTCA |
| USP3-A | Genepharma | GCAGUAGCUACAGUACAUA |
| USP3-B | Genepharma | AGCGCTCTAAGAATCAAGAAA |
| USP4-A | Genepharma | AACATGTCCGAGTTTGTCTGT |
| USP4-B | Genepharma | AACTGTAAGAAGCATCAACAG |
| USP5-A | Genepharma | GGAGAAGUUUGAAUUAGACTT |
| USP5-B | Genepharma | GGAUGUGAAGAUUGUCAUUTT |
| USP6-A | Genepharma | GCACAGUAGCAAACUCAUATT |
| USP6-B | Genepharma | GGGAACUAUUCUACAUCCUTT |
| USP7-A | Genepharma | ACCCUUGGACAAUAUUCCU |
| USP7-B | Genepharma | AGUCGUUCAGUCGUCGUAU |
| USP8-A | Genepharma | CCACAGAUUGAUCGUACUATT |
| USP8-B | Genepharma | GGAUAGGGAACCUUCCAAATT |
| USP9-A | Genepharma | AGAAAUCGCUGGUAUAAAUUU |
| USP9-B | Genepharma | ACACGAUGCUUUAGAAUUUUU |
| USP10-A | Genepharma | GAGAAUCUGUCCAAGGUUAUU |
| USP10-B | Genepharma | GAGGAAAUGUUGAACCUAAUU |
| USP11-A | Genepharma | CCGTGATGATATCTTCGTCTA |
| USP11-B | Genepharma | CCGTGACTACAACAACTCCTA |
| USP12-A | Genepharma | CAGAUCUCUUCCAUAGCAUTT |
| USP12-B | Genepharma | AAACAGACGAAGTTCTAAAGG |
| USP13-A | Genepharma | CCATGTGAACAATGATCCA |
| USP13-B | Genepharma | AAGGGAACATGTTGAAAGACAT |
| USP14-A | Genepharma | GCAUAUCGCUUACGUUCUA |
| USP14-B | Genepharma | GAAACAAGAUGAAUGGAUUUU |
| USP15-A | Genepharma | GGUUGGAAUAAACUUGUCATT |
| USP15-B | Genepharma | GGAACACCTTATTGATGAA |
| USP16-A | Genepharma | UUCUCCAUUGCUCCCUUCC |
| USP16-B | Genepharma | CCUCCUGUUCUUACUCUUCAUUUAA |
| USP17-A | Genepharma | GCAGGAAGATGCCCATGAA |
| USP17-B | Genepharma | GAAAUUCCUUCAAGAGCAA |
| USP18-A | Genepharma | CCACUGGCAGGAAACUGCAUAUCUU |
| USP18-B | Genepharma | ACAUGAAGAUGGAGUGCUATT |
| USP19-A | Genepharma | GGAGGAGATGGCAGTGGCA |
| USP19-B | Genepharma | GAGACAGGGTCTCGATATG |
| USP20-A | Genepharma | GGACAAUGAUGCUCACCUAUU |
| USP20-B | Genepharma | ACCGUCGUACGUGCUCAAGAA |
| USP21-A | Genepharma | GCCGTTCCAAGTCTGTGA |
| USP21-B | Genepharma | AAGATGGCTCATCACACACTC |
| USP22-A | Genepharma | CGAAGGGTACTTGCTGTTCTA |
| USP22-B | Genepharma | TGTGCCAGGACTACATCTATG |
| USP24-A | Genepharma | CUUCUACUGUGGCUUGCUU |
| USP24-B | Genepharma | GGACGAGAAUUGAUAAAGATT |
| USP26-A | Genepharma | GATATCCTGGCTCCACACA |
| USP26-B | Genepharma | CCACAAAGCUGGAGGUAAA |
| USP28-A | Genepharma | CUGCAUUCACCUUAUCAUUTT |
| USP28-B | Genepharma | GTATGGACAAGAGCGTTGG |
| USP29-A | Genepharma | CCCAUCAAGUUUAGAGGAUTT |
| USP29-B | Genepharma | GGAAUAUGCUGAAGGAAAUTT |
| USP30-A | Genepharma | CAAAUUACCTGCCGCACAA |
| USP30-B | Genepharma | CGUCAGAUAUAAAGUCAUG |
| USP32-A | Genepharma | GGACCUGUGGACUCUCAUATT |
| USP32-B | Genepharma | GGUGAUUUACUGUUCUUUUTT |
| USP33-A | Genepharma | GAUCAUGUGGCGAAGCAUATT |
| USP33-B | Genepharma | GGCUUGGAUCUUCAGCCAUTT |
| USP36-A | Genepharma | CGTATATGTCCCAGAATAA |
| USP36-B | Genepharma | GCGGTCAGTCAGGATGCTATT |
| USP37-A | Genepharma | GAUUUGACAGAAUGAGCGA |
| USP37-B | Genepharma | GAGGAUCGAUUAAGACUGU |
| USP42-A | Genepharma | CCCUCUUCUACCAUUACCA |
| USP42-B | Genepharma | GUUAAUAGGUCCUCAGUGA |
| USP44-A | Genepharma | GCATGTGACAACAAATCAA |
| USP44-B | Genepharma | GAACAUGGUUUGAACAAUC |
| USP46-A | Genepharma | CGCTTACCAATGAAACTCGAT |
| USP46-B | Genepharma | GGCAUUGUACUUCUGCCGUTT |
| USP47-A | Genepharma | CAGCAGCATGATGTACAAGAA |
| USP47-B | Genepharma | CTGGATGGAGCACCAAATAAA |
| USP48-A | Genepharma | GGTCATGCATCACGGGAAA |
| USP48-B | Genepharma | GGACTACGTGCTCAATGAT |
| CYLD-A | Genepharma | CGAAGAGGCTGAATCATAA |
| CYLD-B | Genepharma | GAUCGUUCUGUGGGGCAUUTT |
| UCHL1-A | Genepharma | AAGUUAGUCCUAAAGUGUATT |
| UCHL1-B | Genepharma | GCACAAUCGGACUUAUUCATT |
| UCHL3-A | Genepharma | GAAGTTTATGGAGCGCGAC |
| UCHL3-B | Genepharma | GGCACCAAGUAUAGAUGAGTT |
| BAP1-A | Genepharma | GGCTGAGATTGCAAACTATGAG |
| BAP1-B | Genepharma | GGTTTCAGCCCTGAGAGCAAAG |
| UCHL5-A | Genepharma | CAGCAGUUAAUACCACUAGUA |
| UCHL5-B | Genepharma | UCAGAUGUGAUUCGACAAGUA |
| ATXN3-A | Genepharma | TCGGAAGAGACGAGAAGCCTA |
| ATXN3-B | Genepharma | TGCGTCGGTTGTAGGACTAAA |
| ATXN3L-A | Genepharma | GGAAAUAAGGCUUUUAGACTT |
| ATXN3L-B | Genepharma | GCUAGAUGAAGAAGAGAGGTT |
| JOSD1-A | Genepharma | GGUUGUCUCCAAACACCAUTT |
| JOSD1-B | Genepharma | GGCUAUGAAGCUGUUUGGUTT |
| JOSD2-A | Genepharma | UGAUGUCAAUGUGAUCAUGTT |
| JOSD2-B | Genepharma | UGUCAAUGUGAUCAUGGCCTT |
| A20-A | Genepharma | AGACACACGCAACTTTAAA |
| A20-B | Genepharma | CCGAGCTGTTCCACTTGTTAA |
| OTUB1-A | Genepharma | GCAAGUUCUUCGAGCACUU |
| OTUB1-B | Genepharma | CCGACUACCUUGUGGUCUA |
| OTUB2-A | Genepharma | CTTCTGCACTCACGAAGTA |
| OTUB2-B | Genepharma | CCGTTTACCTGCTCTATAA |
| OTUD1-A | Genepharma | CCCAGAGUACGACAACUGGTT |
| OTUD1-B | Genepharma | GGAAUAAGUACCGAUUCCATT |
| OTUD3-A | Genepharma | TCGCAAAGGTCACAAACAA |
| OTUD3-B | Genepharma | GAAAUCAGGGCUUAAAUGA |
| OTUD4-A | Genepharma | GGAAUGGGUAGGACAAGUGTT |
| OTUD4-B | Genepharma | GGUGUUACUGUGUUUUUCATT |
| POH1-A | Genepharma | GAACAAGTCTATATCTCTT |
| POH1-B | Genepharma | ACAGCAGAACAAGTCTATATC |
| BRCC36 A | Genepharma | CCAACAGCATTTGCAGGAATT |
| BRCC36 B | Genepharma | GAGGAAGGACCGAGUAGAATT |
| AMSH-A | Genepharma | UUACAAAUCUGCUGUCAUUUU |
| AMSH-B | Genepharma | CCAGAGTCAGTAGCCATTGTT |
| AMSHLP-A | Genepharma | GCUUGGUUGUAAUAUCACCTT |
| AMSHLP-B | Genepharma | GCUAGAAUCGGAGCAGUUUTT |
| USP25-A | Genepharma | GGGAGUACUUGAAGGUAAATT |

**Supplementary Table 3: shRNA and siRNA sequences**

| **RNAi** | **Target** | **Sequence (5’-3’)** |
| --- | --- | --- |
| siNC-1 | - | UUCUUCGAACGUGUCACGUTT |
| siUSP7-1 | Human USP7 | CGTGGTGTCAAGGTGTACTAA |
| siUSP7-3 | Human USP7 | ACCCUUGGACAAUAUUCCU |
| siUSP7-5 | Human USP7 | GCAUAGUGAUAAACCUGUATT |
| siNC-2 | - | GTTGAAGCATTGCGCAGTATA |
| siDCAF12-2 | Human DCAF12 | AGGAGAGAGAGUUUCACCUTT |
| siDCAF12-3 | Human DCAF12 | CAUAUUGCCGUGAGAAUGUTT |
| shNC | - | TTCTCCGAACGTGTCACGT |
| shUSP7 | Human USP7 | ACCCTTGGACAATATTCCT |

**Supplementary Table 4: Human DUBs plasmid Library (33 DUBs)**

| **Plasmid Name** | **Source** | **Catalog number #** |
| --- | --- | --- |
| Flag-HA-USP1 | Addgene | 22596 |
| Flag-HA-USP3 | Addgene | 22582 |
| Flag-HA-USP5 | Addgene | 22590 |
| pQFlag-USP7 WT puroR | Addgene | 46751 |
| Flag-HA-USP8 | Addgene | 22608 |
| Flag-HA-USP11 | Addgene | 22566 |
| Flag-HA-USP13 | Addgene | 22568 |
| Flag-HA-USP14 | Addgene | 22569 |
| Flag-HA-USP15 | Addgene | 22570 |
| Flag-HA-USP16 | Addgene | 22595 |
| Flag-HA-DUB3 | Addgene | 22593 |
| Flag-HA-USP18 | Addgene | 22572 |
| pRK-flag-USP19 | Addgene | 78597 |
| Flag-HA-USP20 | Addgene | 22573 |
| Flag-HA-USP22 | Addgene | 22575 |
| Flag-HA-USP26 | Addgene | 22598 |
| pDZ Flag USP28 | Addgene | 15665 |
| Flag-HA-USP29 | Addgene | 22599 |
| Flag-HA-USP39 | Addgene | 22581 |
| Flag-HA-USP46 | Addgene | 22584 |
| Flag-HA-USP52 | Addgene | 22589 |
| Flag-HA-USP53 | Addgene | 22606 |
| Flag-HA-CYLD | Addgene | 22544 |
| Flag-HA-OTUB1 | Addgene | 22551 |
| Flag-HA-OTUB2 | Addgene | 22552 |
| Flag-HA-BAP1 | Addgene | 22539 |
| Flag-HA-JOSD2 | Addgene | 22548 |
| Flag-HA-OTUD1 | Addgene | 22553 |
| Flag-HA-OTUD5 | Addgene | 22610 |
| Flag-HA-OTUD6B | Addgene | 22555 |
| Flag-HA-YOD1 | Addgene | 22554 |
| Flag-HA-UCHL1 | Addgene | 22563 |
| Flag-HA-USP37 | Addgene | 22602 |

**Supplementary Table 5: Antibodies used**

| **Specificity** | **Source** | **Catalog number** | **Application (Concentration)** |
| --- | --- | --- | --- |
| TAZ | CST | #4883 | WB(1:1000), IP(1μg) |
| TAZ | CST | #83669 | IF(1:200), IHC(1:200) |
| USP7 | Bethyl Laboratories | A300-033A-M | WB(1:1000), IHC(1:200), IP(1μg) |
| USP7 | GeneTex | GTX631108 | IF(1:400) |
| DYKDDDDK (Flag-tag) | Proteintech | 66008-3-Ig | WB(1:1000), IP(1μg) |
| Myc-tag | Proteintech | 16286-1-AP | WB(1:5000), IP(1μg) |
| HA-tag | Proteintech | 51064-5-AP | WB(1:5000) |
| V5-tag | Proteintech | 14440-1-AP | WB(1:10000) |
| Ki-67 | Proteintech | 27309-1-AP | IHC(1:200) |
| Cleaved Caspase3 | CST | #9661 | IHC(1:200) |
| CYR61 | CST | #14479 | WB(1:1000) |
| E-cadherin | CST | #14472 | WB(1:1000) |
| N-cadherin | CST | #13116 | WB(1:1000) |
| Vimentin | Proteintech | 10366-1-AP | WB(1:3000) |
| MST1 | CST | #3682 | WB(1:1000) |
| MST2 | CST | #3952 | WB(1:1000) |
| LATS1 | CST | #3477 | WB(1:1000) |
| LATS2 | CST | #5888 | WB(1:1000) |
| Phospho-TAZ (Ser89) | CST | #59971 | WB(1:1000) |
| YAP | CST | #14074 | WB(1:1000) |
| Phospho-YAP (Ser127) | CST | #13008 | WB(1:1000) |
| Pan-TEAD | CST | #13295 | WB(1:1000) |
| TEAD4 | Proteintech | ab197589 | WB(1:1000), IP(1μg) |
| Lamin A/C | Proteintech | 10289-1-AP | WB(1:5000) |
| GAPDH | CST | #2118 | WB (1:2000) |

**Supplementary Table 6: qPCR primer sequences**

| **Target (human)** | **Forward (5’-3’)** | **Reverse (5’-3’)** |
| --- | --- | --- |
| TAZ/WWTR1 | GGCTGGGAGATGACCTTCAC | AGGCACTGGTGTGGAACTGAC |
| USP7 | CCCTCCGTGTTTTGTGCGA | AGACCATGACGTGGAATCAGA |
| CYR61/CCN1 | GCCACAAGCTGTCCAGTCTAATCG | TGCATTCTCCAGCCATCAAGAGAC |
| E-Cadherin/CDH1 | ATTTTTCCCTCGACACCCGAT | TCCCAGGCGTAGACCAAGA |
| N-Cadherin/CDH2 | TGCGGTACAGTGTAACTGGG | GAAACCGGGCTATCTGCTCG |
| Vimentin | GACGCCATCAACACCGAGTT | CTTTGTCGTTGGTTAGCTGGT |
| DCAF12 | ATGGCCCGGAAAGTAGTTAGC | ACTTCCCGGTTCTTCAAGTAGT |
| 18S rRNA | ACACGGACAGGATTGACAGA | GGACATCTAAGGGCATCACA |
