# Supplementary material for: The deubiquitinase USP7 promotes HNSCC progression via deubiquitinating and stabilizing TAZ
Source: Cell Death Dis. 2022 Aug 5;13(8):677. doi: 10.1038/s41419-022-05113-z (PMC9356134; doi:10.1038/s41419-022-05113-z)
Supplement: Supplementary file 17 — Original Western Blots [file 41419_2022_5113_MOESM17_ESM.pdf]

Fig1B

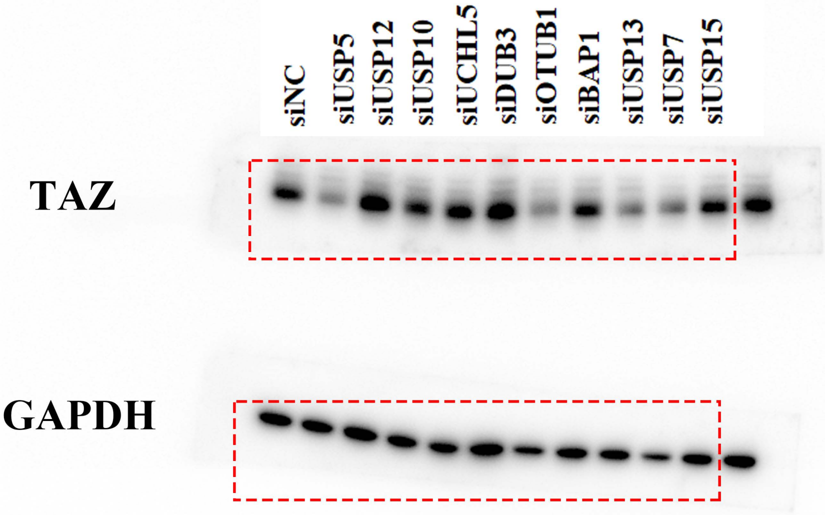
siNC
siUSP5
siUSP12
siUSP10
siUCHL5
siDUB3
siOTUB1
siBAP1
siUSP13
siUSP7
siUSP15
TAZ
GAPDH

Fig1D

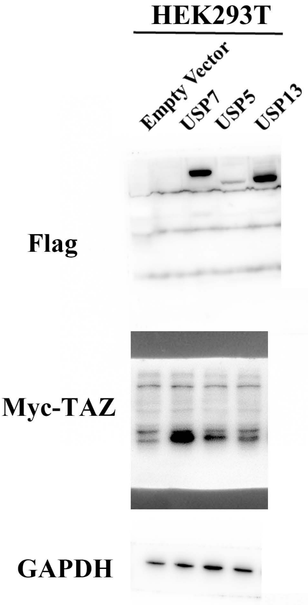

Fig1E

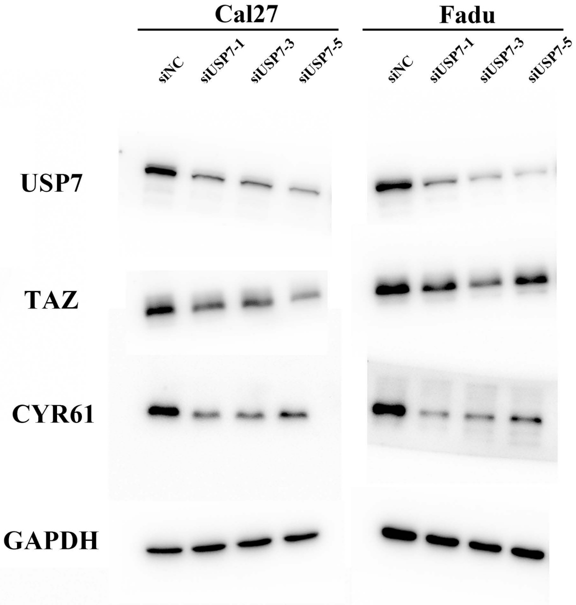

Fig1F

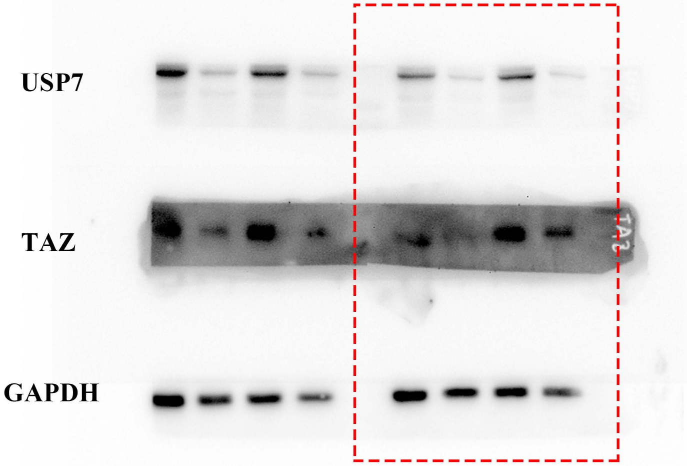

Fig1G

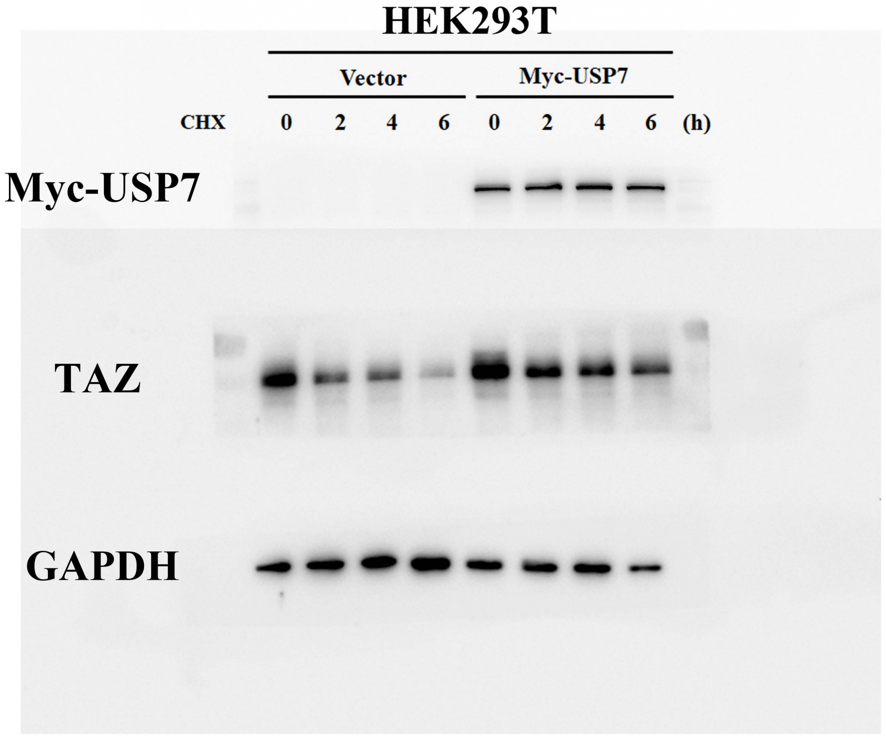

Fig1H

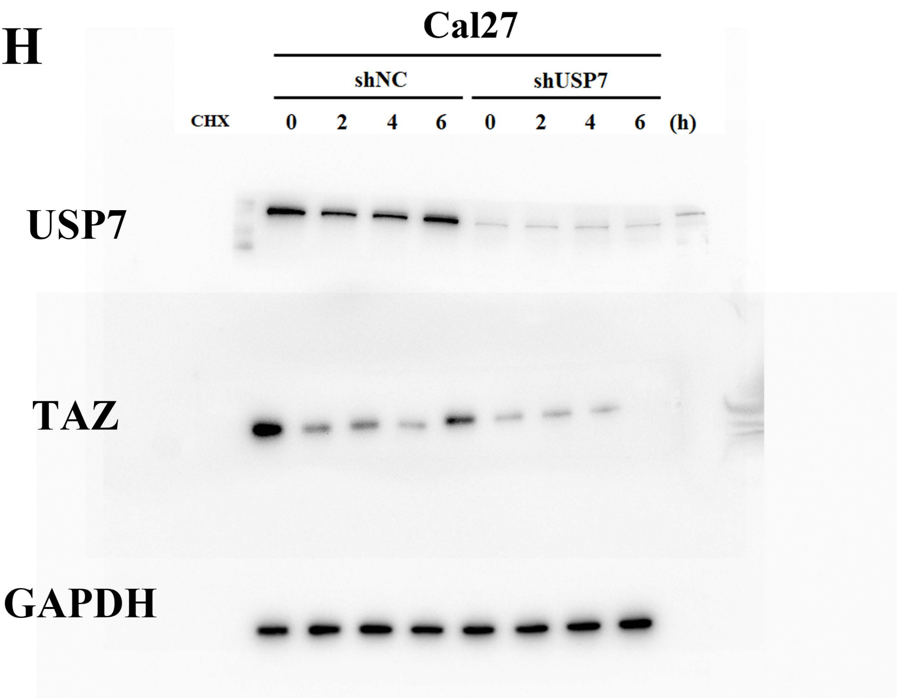

Fig1I

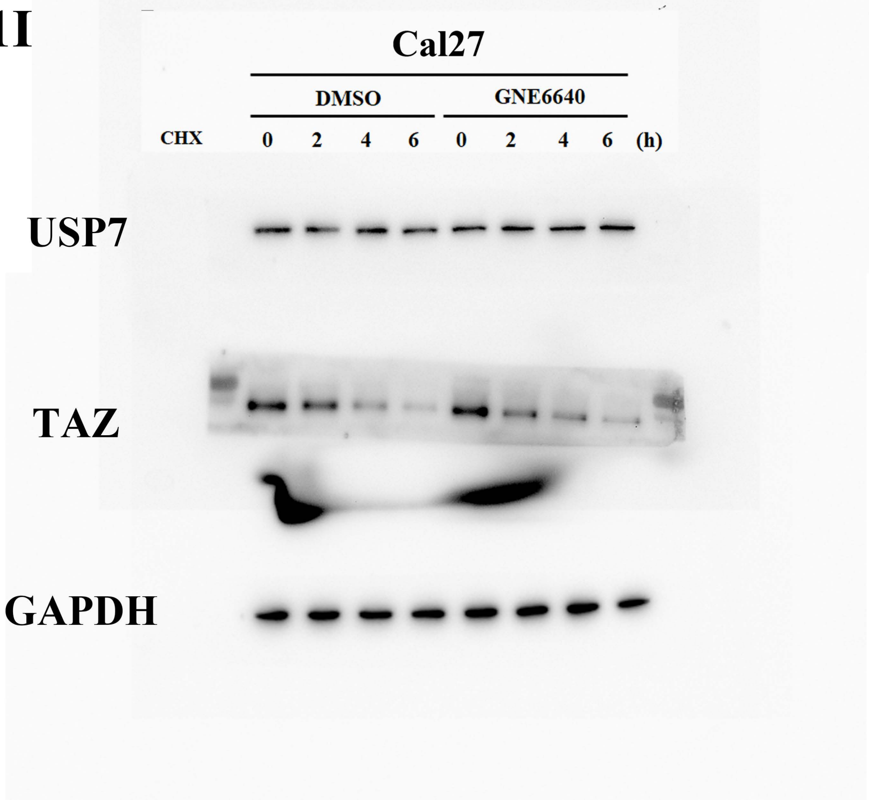

Fig3A

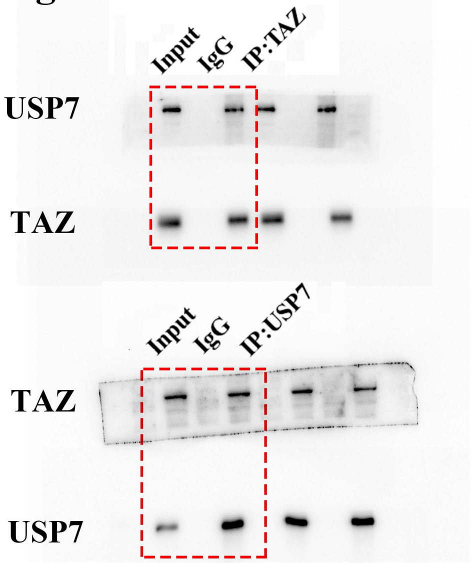

Fig3B

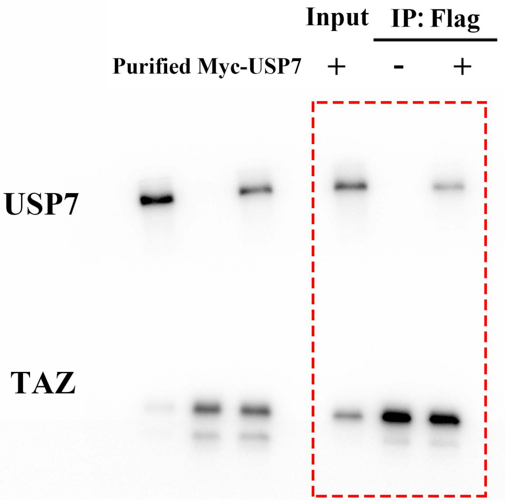

Fig3D

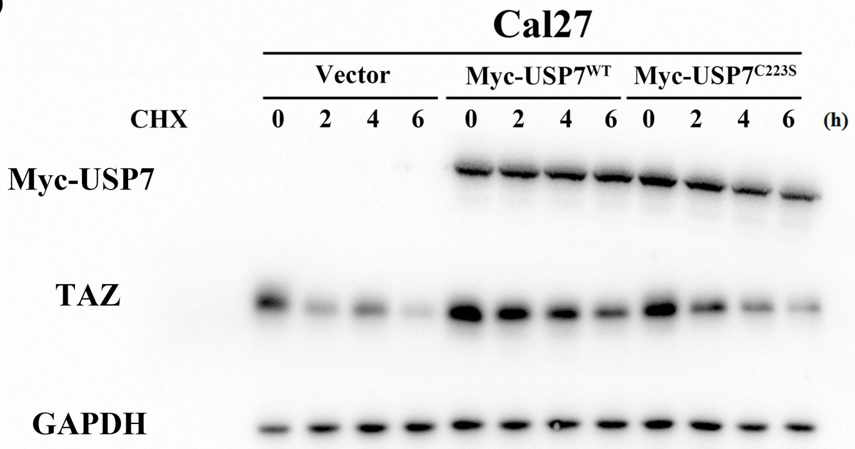

Fig3E

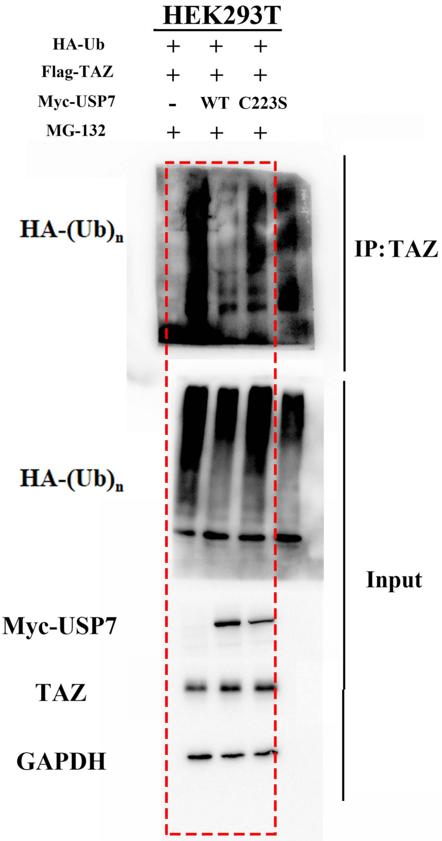
HEK293T
HA-Ub + + +
Flag-TAZ + + +
Myc-USP7 - WT C223S
MG-132 + + +
HA-(Ub)n
IP:TAZ
HA-(Ub)n
Myc-USP7
TAZ
GAPDH
Input

Fig3F

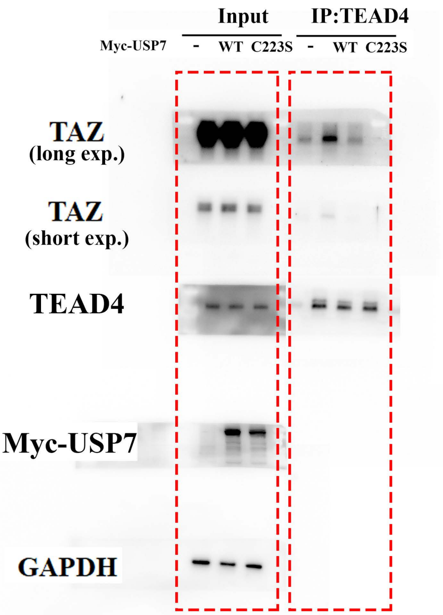
Input
IP:TEAD4
Myc-USP7 - WT C223S - WT C223S
TAZ
(long exp.)
TAZ
(short exp.)
TEAD4
Myc-USP7
GAPDH

Fig3G

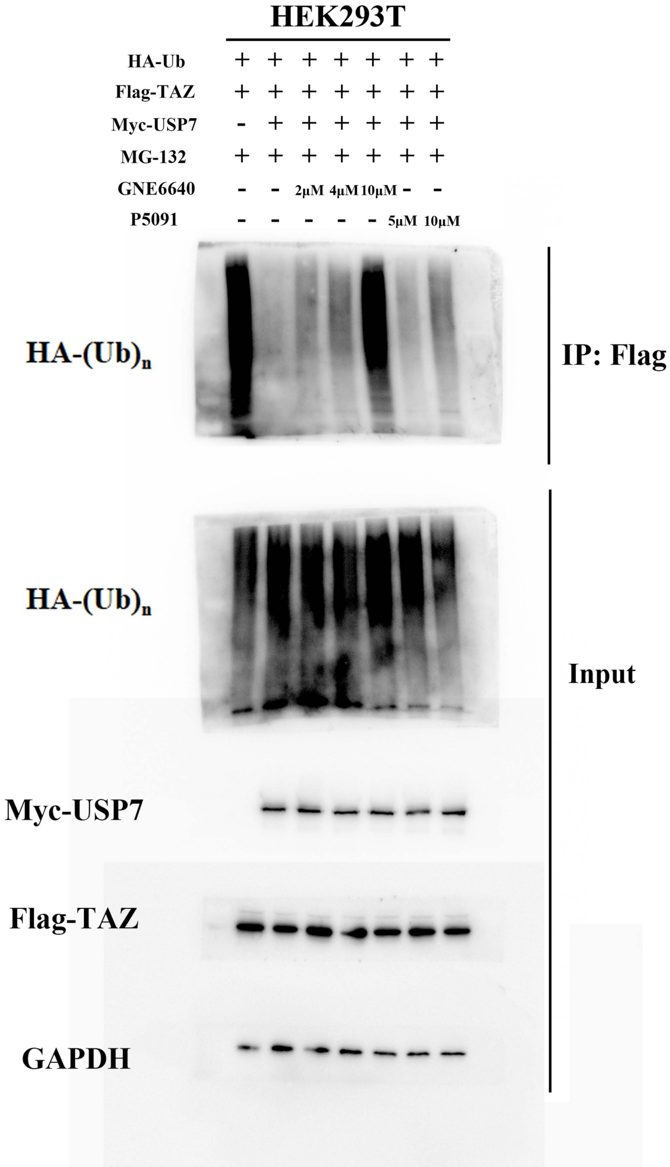

HEK293T
HA-Ub + + + + + + +
Flag-TAZ + + + + + + +
Myc-USP7 - + + + + + +
MG-132 + + + + + + +
GNE6640 - - 2μM 4μM 10μM - -
P5091 - - - - - 5μM 10μM
HA-(Ub)n
IP: Flag
HA-(Ub)n
Input
Myc-USP7
Flag-TAZ
GAPDH

Fig3H

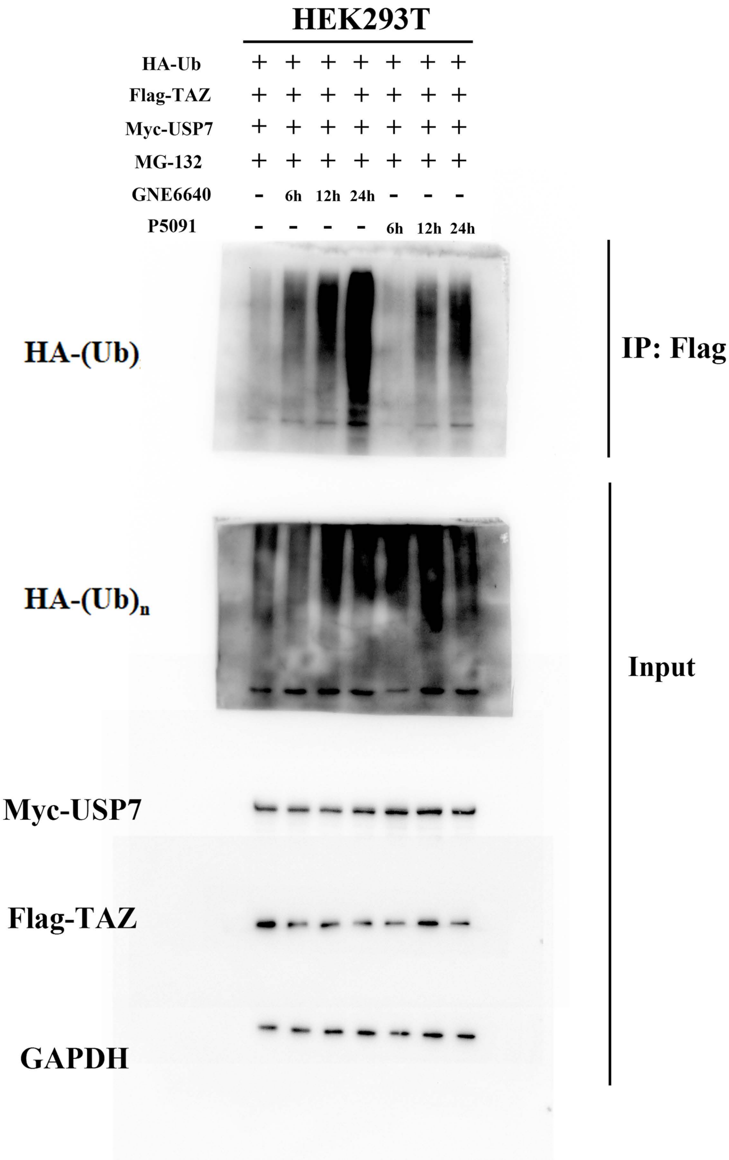

HEK293T
HA-Ub + + + + + + +
Flag-TAZ + + + + + + +
Myc-USP7 + + + + + + +
MG-132 + + + + + + +
GNE6640 - 6h 12h 24h - - -
P5091 - - - - 6h 12h 24h
HA-(Ub)
IP: Flag
HA-(Ub)n
Input
Myc-USP7
Flag-TAZ
GAPDH

Fig4B

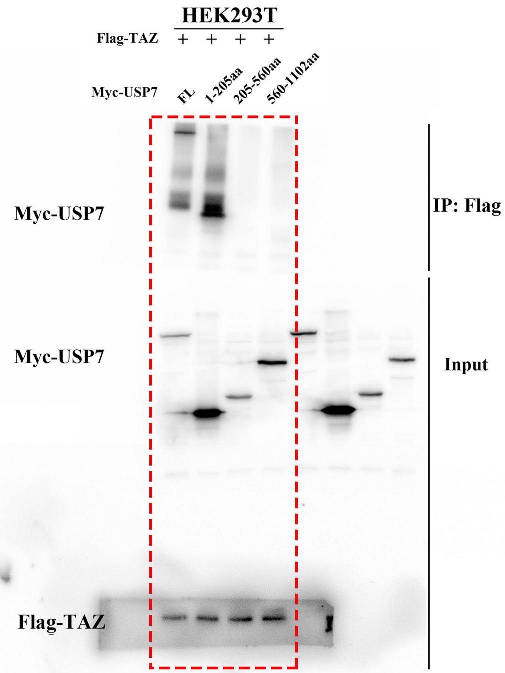
HEK293T
Flag-TAZ + + + +
Myc-USP7 FL 1-205aa 205-560aa 560-1102aa
Myc-USP7
IP: Flag
Myc-USP7
Input
Flag-TAZ

Fig4C

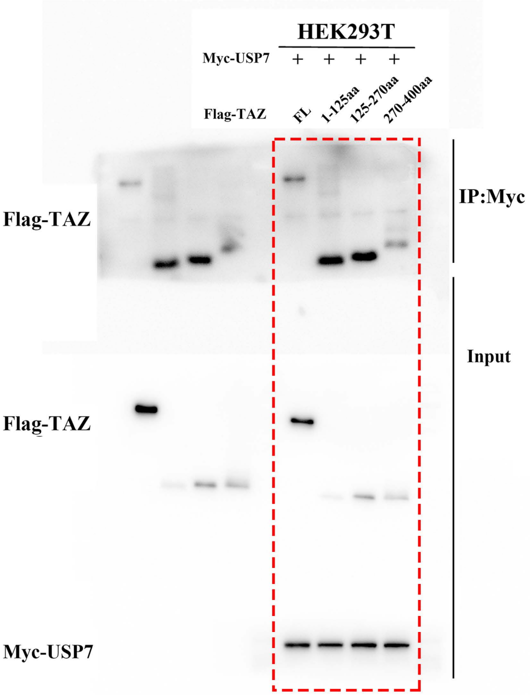
HEK293T
Myc-USP7 + + + +
Flag-TAZ FL 1-125aa 125-270aa 270-400aa
Flag-TAZ
IP:Myc
Flag-TAZ
Input
Myc-USP7

Fig4D

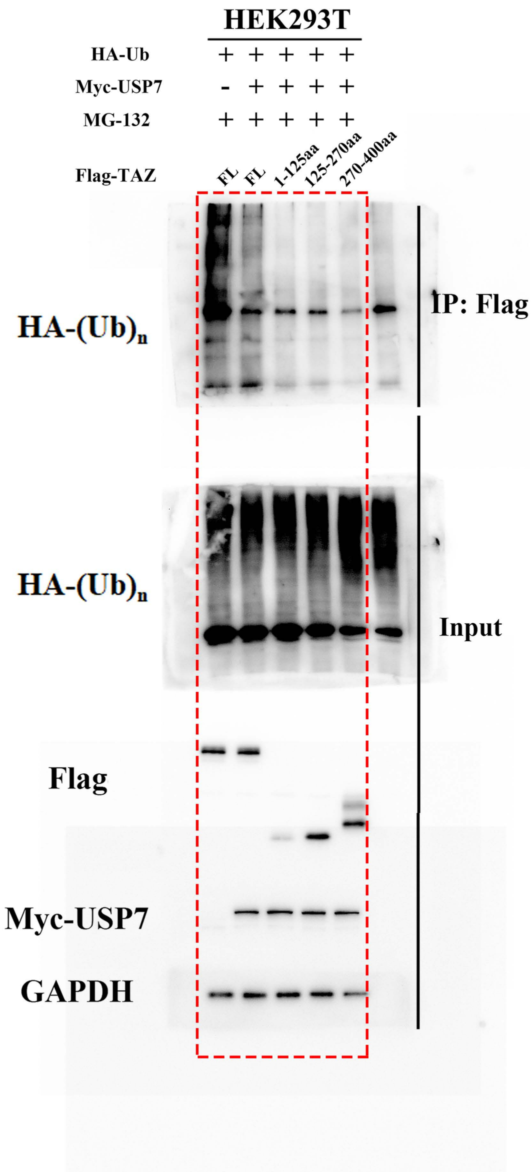
HEK293T
HA-Ub + + + + +
Myc-USP7 - + + + +
MG-132 + + + + +
Flag-TAZ FL FL 1-125aa 125-270aa 270-400aa
HA-(Ub)n
IP: Flag
HA-(Ub)n
Input
Flag
Myc-USP7
GAPDH

Fig4E

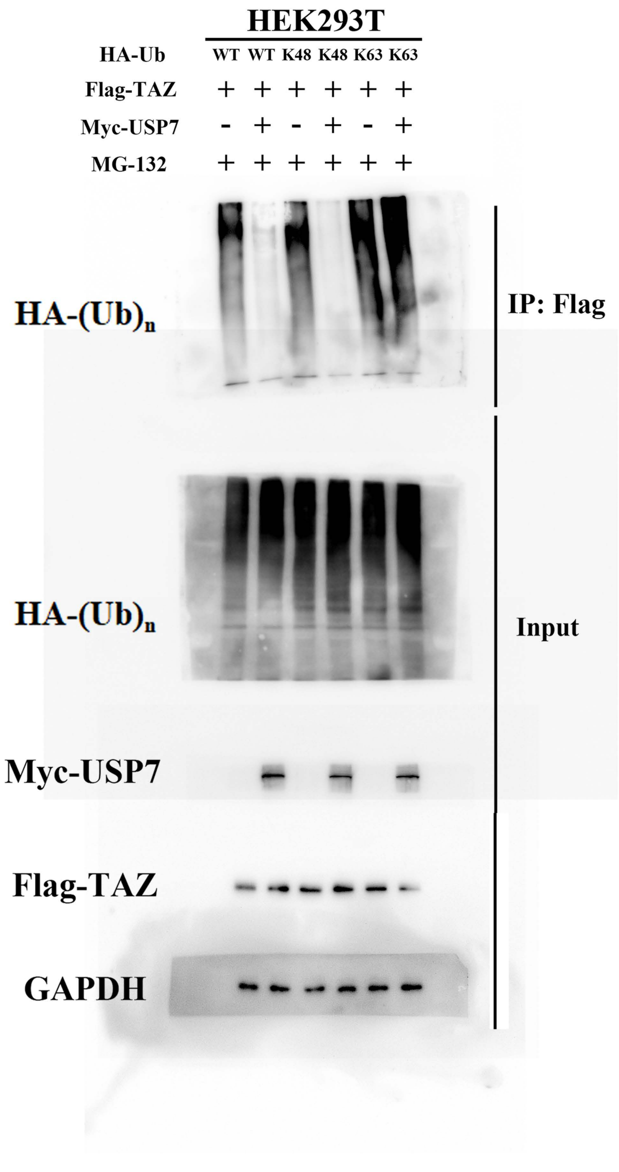
HEK293T
HA-Ub WT WT K48 K48 K63 K63
Flag-TAZ + + + + + +
Myc-USP7 - + - + - +
MG-132 + + + + + +
HA-(Ub)n
IP: Flag
HA-(Ub)n
Input
Myc-USP7
Flag-TAZ
GAPDH

Fig4F

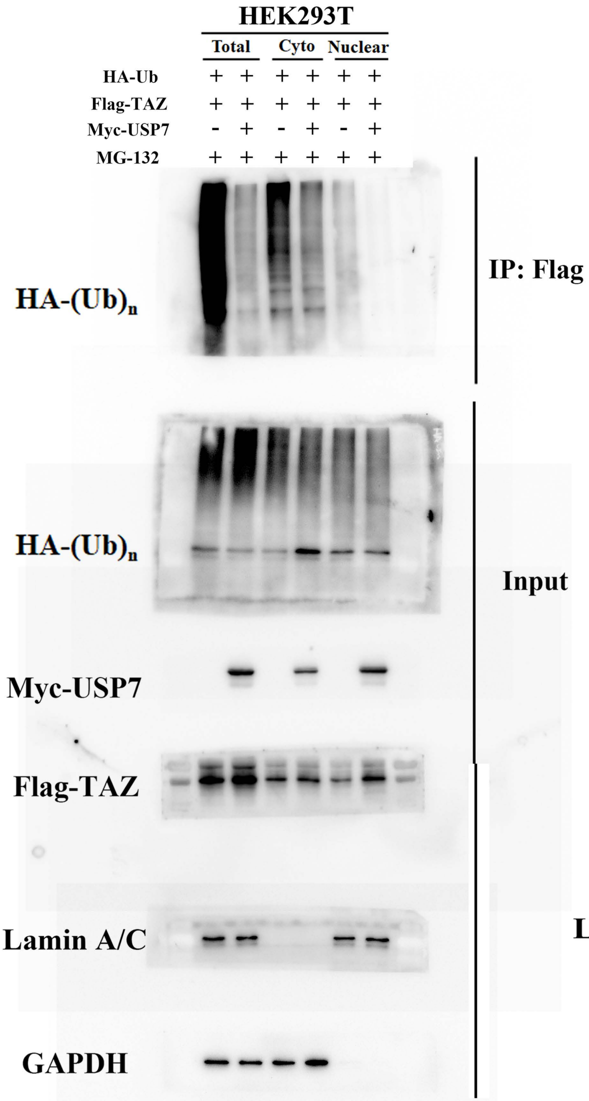
HEK293T
Total
Cyto
Nuclear
HA-Ub + + + + + +
Flag-TAZ + + + + + +
Myc-USP7 - + - + - +
MG-132 + + + + + +
HA-(Ub)$_n$
IP: Flag
HA-(Ub)$_n$
Input
Myc-USP7
Flag-TAZ
Lamin A/C
GAPDH

Fig4G

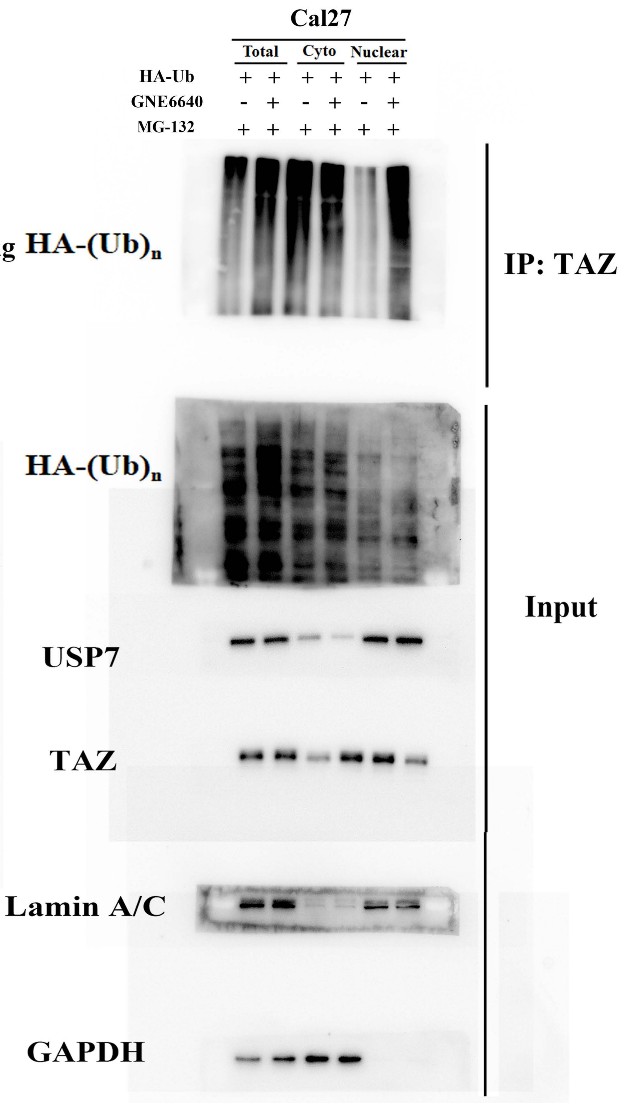
Cal27
Total
Cyto
Nuclear
HA-Ub + + + + + +
GNE6640 - + - + - +
MG-132 + + + + + +
HA-(Ub)$_n$
IP: TAZ
HA-(Ub)$_n$
Input
USP7
TAZ
Lamin A/C
GAPDH

Fig5A

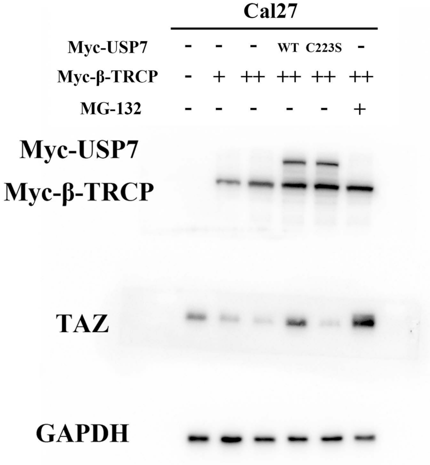
Cal27
Myc-USP7 - - - WT C223S -
Myc-β-TRCP - + ++ ++ ++ ++
MG-132 - - - - - +
Myc-USP7
Myc-β-TRCP
TAZ
GAPDH

Fig5B

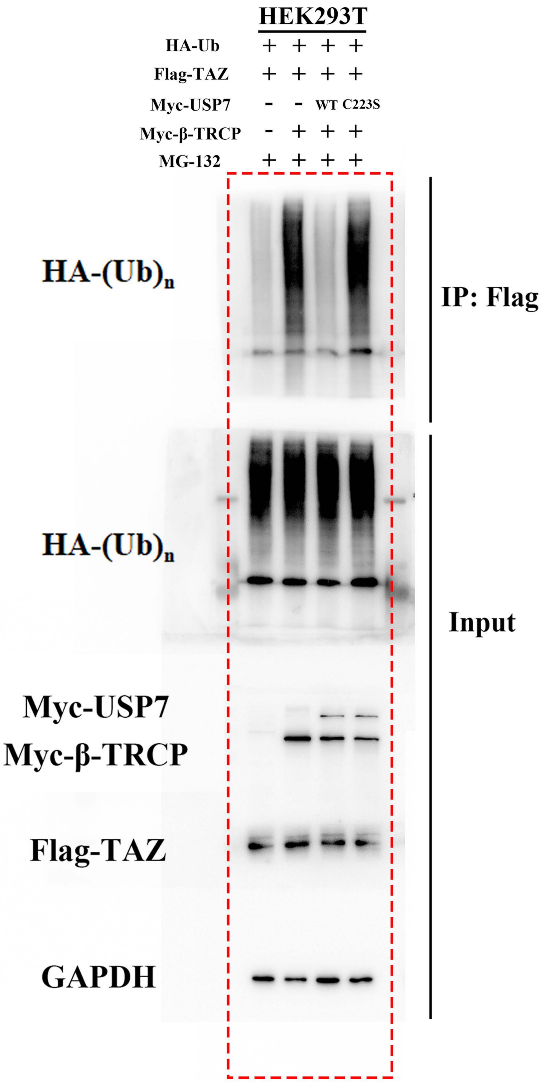
HEK293T
HA-Ub + + + +
Flag-TAZ + + + +
Myc-USP7 - - WT C223S
Myc-β-TRCP - + + +
MG-132 + + + +
HA-(Ub)n
IP: Flag
HA-(Ub)n
Input
Myc-USP7
Myc-β-TRCP
Flag-TAZ
GAPDH

Fig5C

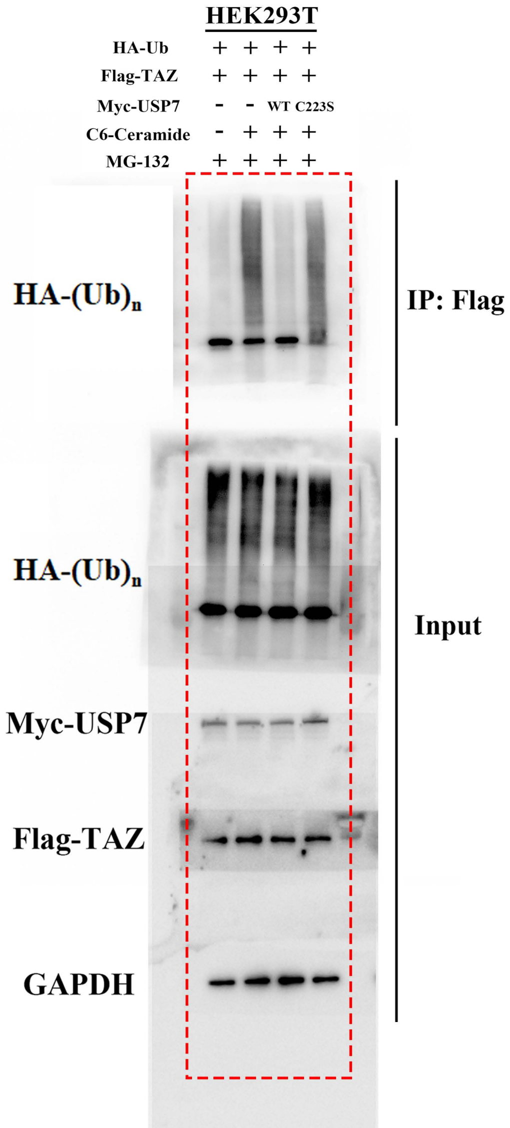
HEK293T
HA-Ub + + + +
Flag-TAZ + + + +
Myc-USP7 - - WT C223S
C6-Ceramide - + + +
MG-132 + + + +
HA-(Ub)n
IP: Flag
HA-(Ub)n
Input
Myc-USP7
Flag-TAZ
GAPDH

Fig5D

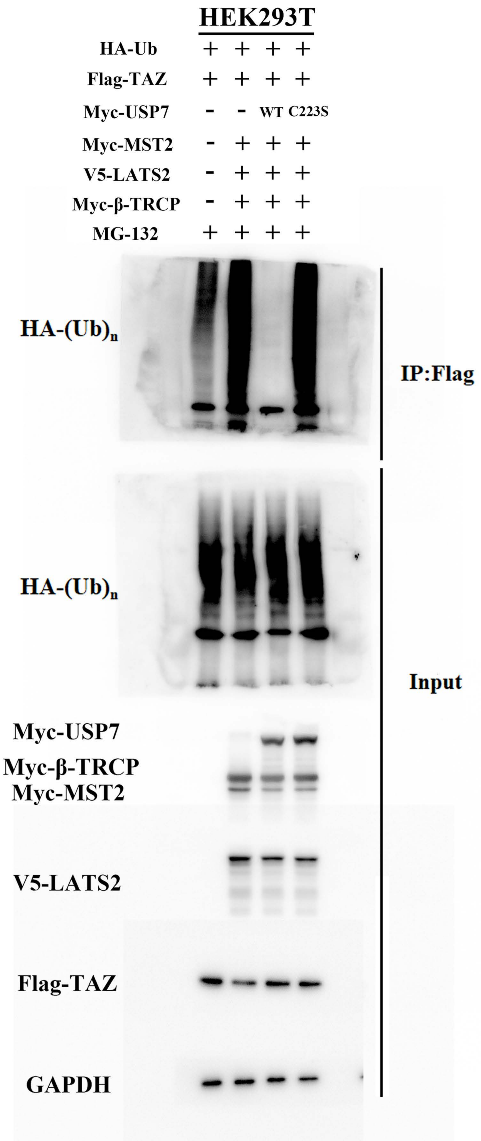
HEK293T
HA-Ub + + + +
Flag-TAZ + + + +
Myc-USP7 - - WT C223S
Myc-MST2 - + + +
V5-LATS2 - + + +
Myc-β-TRCP - + + +
MG-132 + + + +
HA-(Ub)n
IP:Flag
HA-(Ub)n
Input
Myc-USP7
Myc-β-TRCP
Myc-MST2
V5-LATS2
Flag-TAZ
GAPDH

Fig5F

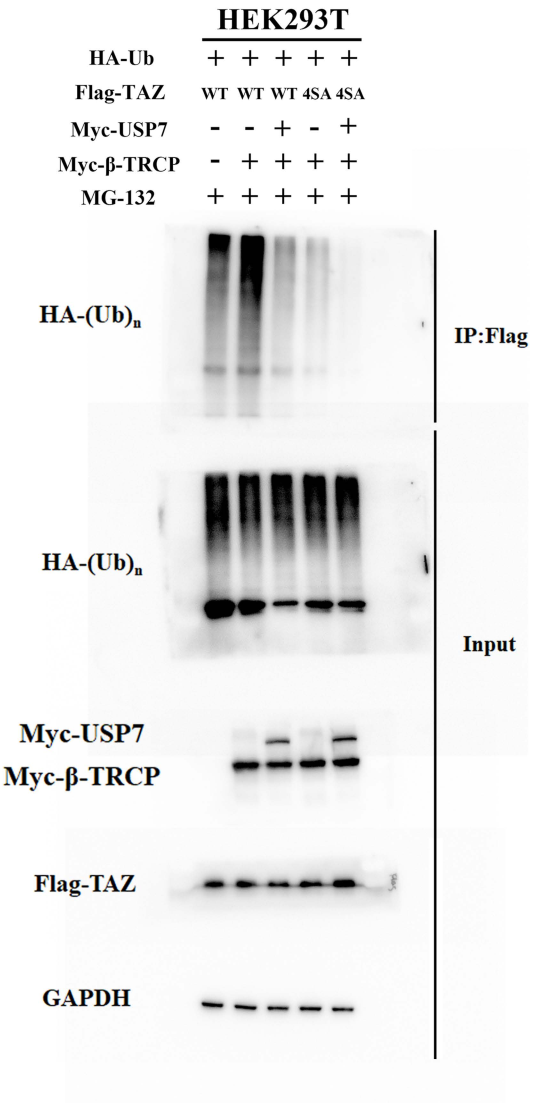
HEK293T
HA-Ub + + + + +
Flag-TAZ WT WT WT 4SA 4SA
Myc-USP7 - - + - +
Myc-β-TRCP - + + + +
MG-132 + + + + +
HA-(Ub)n
IP:Flag
HA-(Ub)n
Input
Myc-USP7
Myc-β-TRCP
Flag-TAZ
GAPDH

Fig6B

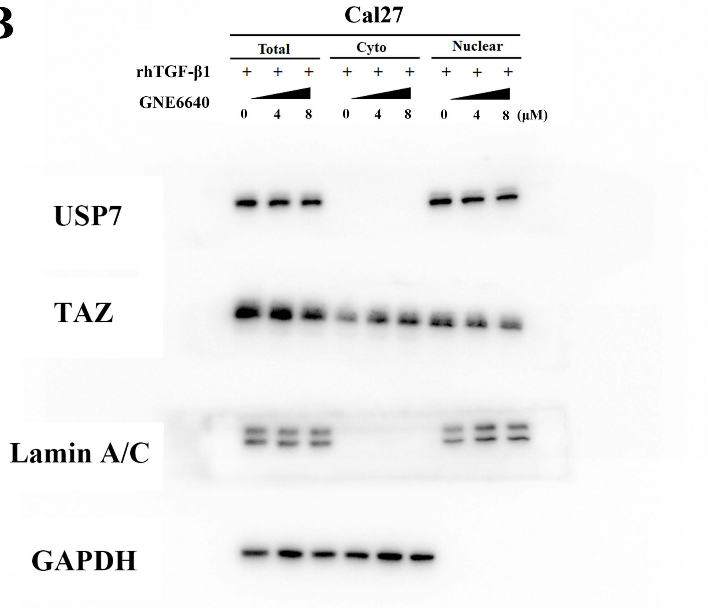
Cal27
Total
Cyto
Nuclear
rhTGF-β1
GNE6640
0 4 8 0 4 8 0 4 8 (μM)
USP7
TAZ
Lamin A/C
GAPDH

Fig6D

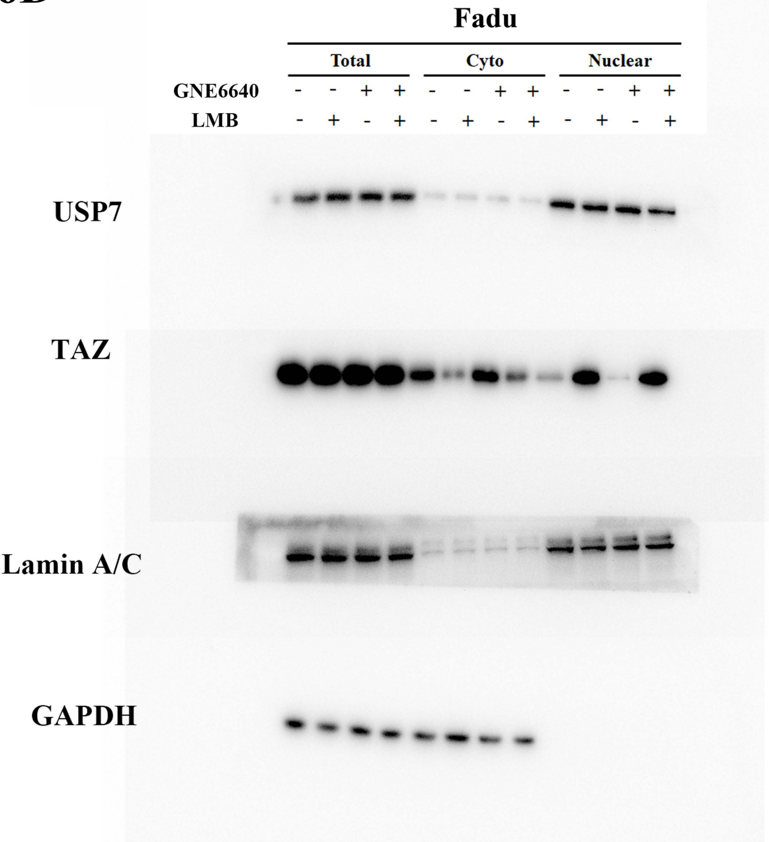
Fadu
Total
Cyto
Nuclear
GNE6640 - - + + - - + + - - + +
LMB - + - + - + - + - + - +
USP7
TAZ
Lamin A/C
GAPDH

Fig7F

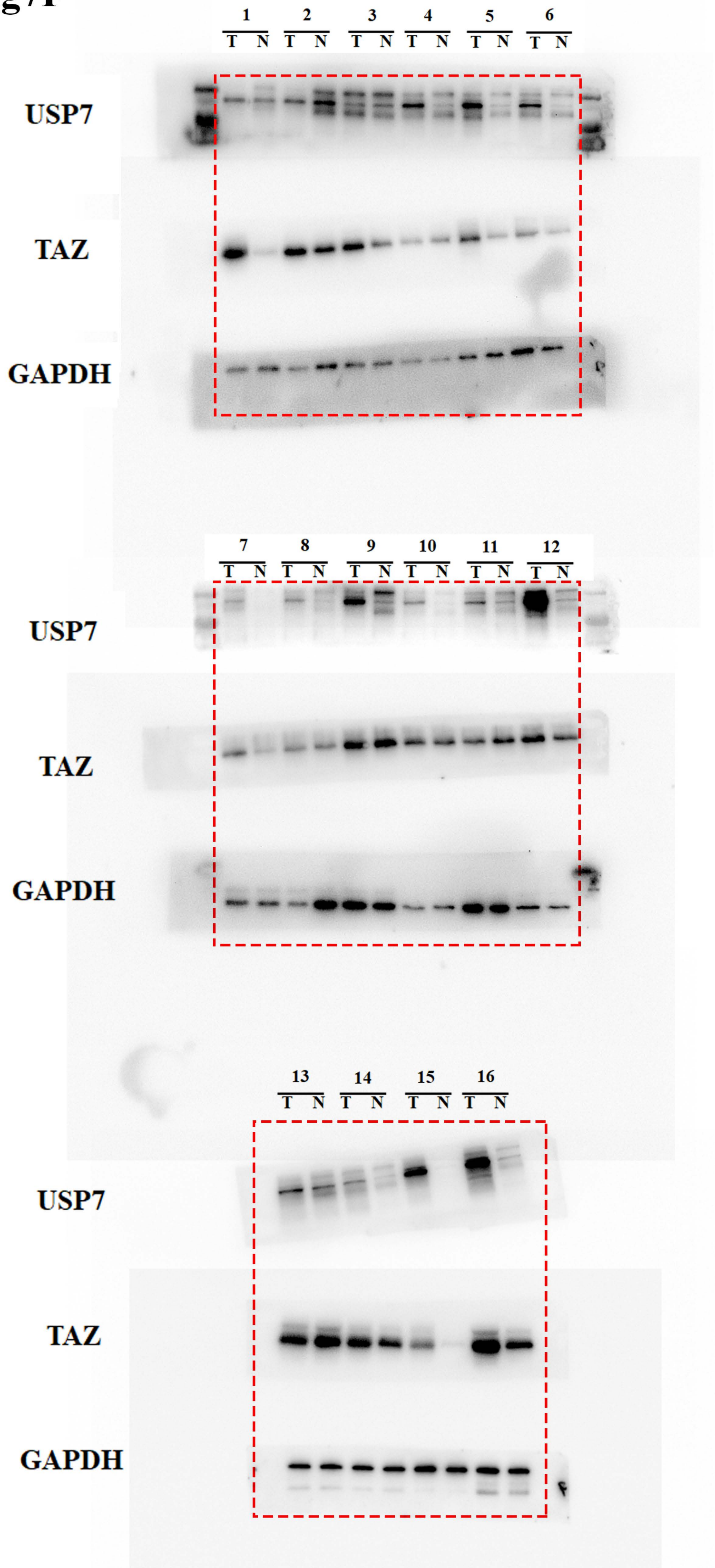

1 2 3 4 5 6
T N T N T N T N T N T N
USP7
TAZ
GAPDH
7 8 9 10 11 12
T N T N T N T N T N T N
USP7
TAZ
GAPDH
13 14 15 16
T N T N T N T N
USP7
TAZ
GAPDH
