# Supplementary material for: The deubiquitinase USP7 promotes HNSCC progression via deubiquitinating and stabilizing TAZ
Source: Cell Death Dis. 2022 Aug 5;13(8):677. doi: 10.1038/s41419-022-05113-z (PMC9356134; doi:10.1038/s41419-022-05113-z)
Supplement: Supplementary file 18 — Author Contribution Statement [file 41419_2022_5113_MOESM18_ESM.docx]

**Authors' contributions**

Dr. Jin Li performed most experiments, data collection and analysis and manuscript writing. Drs. Yibin Dai, Han Ge, Songsong Guo conducted animal experiments, histological and statistical analyses. Drs Wei Zhang, Yanling Wang and Prof. Laikui Liu performed histopathological analyses, patient follow-up and data collection. Profs. Jie Cheng and Hongbing Jiang conceived and supervised the whole project. All authors have read and approved the final manuscript.
